# Estimated Effectiveness of 2024-2025 COVID-19 Vaccination Against Severe COVID-19

**DOI:** 10.1001/jamanetworkopen.2025.57415

**Published:** 2026-02-03

**Authors:** Kevin C. Ma, Alexander Webber, Adam S. Lauring, Emily Bendall, Leigh K. Papalambros, Basmah Safdar, Adit A. Ginde, Ithan D. Peltan, Samuel M. Brown, Manjusha Gaglani, Shekhar Ghamande, Cristie Columbus, Nicholas M. Mohr, Kevin W. Gibbs, David N. Hager, Matthew E. Prekker, Michelle N. Gong, Amira Mohamed, Nicholas J. Johnson, Akram Khan, Catherine L. Hough, Abhijit Duggal, Jennifer G. Wilson, Nida Qadir, Steven Y. Chang, Christopher Mallow, Laurence W. Busse, Jennie H. Kwon, Matthew C. Exline, Ivana A. Vaughn, Mayur Ramesh, Jarrod M. Mosier, Aleda M. Leis, Estelle S. Harris, Adrienne Baughman, Sydney A. Cornelison, Paul W. Blair, Cassandra A. Johnson, Nathaniel M. Lewis, Sascha Ellington, Todd W. Rice, Carlos G. Grijalva, H. Keipp Talbot, Jonathan D. Casey, Natasha Halasa, James D. Chappell, Yuwei Zhu, Wesley H. Self, Fatimah S. Dawood, Diya Surie

**Affiliations:** 1 Coronavirus and Other Respiratory Viruses Division, National Center for Immunization and Respiratory Diseases, Centers for Disease Control and Prevention, Atlanta, Georgia; 2 Department of Internal Medicine, University of Michigan, Ann Arbor; 3 Department of Microbiology and Immunology, University of Michigan, Ann Arbor; 4 Department of Emergency Medicine, Yale University School of Medicine, New Haven, Connecticut; 5 Department of Emergency Medicine, University of Colorado School of Medicine, Aurora; 6 Department of Pulmonary/Critical Care Medicine, Intermountain Medical Center, Murray, Utah; 7 Division of Respiratory, Critical Care, and Occupational Pulmonary Medicine, Department of Medicine, University of Utah, Salt Lake City; 8 Baylor Scott and White Health, Temple, Texas; 9 Baylor College of Medicine, Temple, Texas; 10 Texas A&M University College of Medicine, Dallas; 11 Department of Emergency Medicine, University of Iowa, Iowa City; 12 Department of Medicine, Wake Forest School of Medicine, Winston-Salem, North Carolina; 13 Department of Medicine, Johns Hopkins University School of Medicine, Baltimore, Maryland; 14 Department of Emergency Medicine, Hennepin County Medical Center, Minneapolis, Minnesota; 15 Department of Medicine, Montefiore Medical Center, Albert Einstein College of Medicine, Bronx, New York; 16 Department of Emergency Medicine, University of Washington, Seattle; 17 Division of Pulmonary, Critical Care and Sleep Medicine, University of Washington, Seattle; 18 Department of Medicine, Oregon Health and Sciences University, Portland; 19 Department of Medicine, Cleveland Clinic, Cleveland, Ohio; 20 Department of Emergency Medicine, Stanford University School of Medicine, Stanford, California; 21 Department of Medicine, University of California, Los Angeles; 22 Department of Medicine, University of Miami, Miami, Florida; 23 Department of Medicine, Emory University School of Medicine, Atlanta, Georgia; 24 Department of Medicine, Washington University in St Louis, St Louis, Missouri; 25 Department of Medicine, The Ohio State University, Columbus; 26 Department of Public Health Sciences, Henry Ford Health, Detroit, Michigan; 27 Division of Infectious Diseases, Henry Ford Health, Detroit, Michigan; 28 Department of Emergency Medicine, University of Arizona, Tucson; 29 Department of Epidemiology, University of Michigan, Ann Arbor; 30 Department of Medicine, University of Utah, Salt Lake City; 31 Department of Emergency Medicine, Vanderbilt University Medical Center, Nashville, Tennessee; 32 Department of Biostatistics, Vanderbilt University Medical Center, Nashville, Tennessee; 33 Vanderbilt Institute for Clinical and Translational Research, Vanderbilt University Medical Center, Nashville, Tennessee; 34 Influenza Division, National Center for Immunization and Respiratory Diseases, Centers for Disease Control and Prevention, Atlanta, Georgia; 35 Department of Medicine, Vanderbilt University Medical Center, Nashville, Tennessee; 36 Department of Health Policy, Vanderbilt University Medical Center, Nashville, Tennessee; 37 Department of Pediatrics, Vanderbilt University Medical Center, Nashville, Tennessee

## Abstract

**Importance:**

As SARS-CoV-2 JN.1 lineage descendants continue to evolve, evaluating COVID-19 vaccine effectiveness (VE) against severe COVID-19 remains important to guide vaccination strategies.

**Objective:**

To estimate the VE of the 2024-2025 COVID-19 vaccines against COVID-19–associated hospitalization and severe in-hospital outcomes overall and by time since dose (7-89, 90-179, and ≥180 days), JN.1 descendant lineage (KP.3.1.1, XEC, LP.8.1), and spike protein mutations associated with immune evasion.

**Design, Setting, and Participants:**

This multicenter, test-negative, case-control study conducted by the Investigating Respiratory Viruses in the Acutely Ill Network included adult patients (aged ≥18 years) hospitalized between September 1, 2024, and April 30, 2025, at 26 hospitals in 20 US states. Case patients presented with COVID-19–like illness and positive SARS-CoV-2 nucleic acid or antigen test results; control patients had COVID-19–like illness but tested negative for SARS-CoV-2.

**Exposure:**

Receipt of a 2024-2025 COVID-19 vaccine at least 7 days before illness onset.

**Main Outcomes and Measures:**

Main outcomes were COVID-19–associated hospitalization and severe in-hospital outcomes (supplemental oxygen therapy, acute respiratory failure, intensive care unit admission, and invasive mechanical ventilation or death). Logistic regression was used to estimate the odds of vaccination in case and control patients, adjusting for demographics, clinical characteristics, and enrollment region. The VE was estimated as (1 – adjusted odds ratio) × 100%.

**Results:**

A total of 8493 patients (median [IQR] age, 66 [54-76] years; 4338 female [51.1%]), including 1888 case patients with COVID-19 (among whom 951 [50.4%] had successful whole-genome sequencing, including 348 [36.6%] with KP.3.1.1, 218 [22.9%] with XEC, and 134 [14.1%] with LP.8.1 infections) and 6605 control patients were enrolled. Vaccine effectiveness against COVID-19–associated hospitalization was 40% (95% CI, 27%-51%), and protection was sustained through 90 to 179 days after vaccination. Vaccine effectiveness was higher against the most severe outcome of invasive mechanical ventilation or death at 79% (95% CI, 55%-92%). It was 49% (95% CI, 25%-67%) against hospitalization with KP.3.1.1, 34% (95% CI, 4%-56%) against XEC, and 24% (95% CI, −19% to 53%) against LP.8.1, with increasing median time since dose receipt among vaccinated case patients due to sequential circulation patterns (60, 89, and 141 days, respectively). The VE was similar against lineages with spike protein S31 deletion (41% [95% CI, 22%-56%]) and T22N and F59S substitutions (37% [95% CI, 9%-57%]).

**Conclusions and Relevance:**

In this multicenter, case-control analysis of VE, 2024-2025 COVID-19 vaccines may have provided protection against hospitalizations and severe in-hospital outcomes as multiple JN.1 descendant lineages circulated. Monitoring COVID-19 VE, including stratifying by SARS-CoV-2 lineage and spike protein mutations, remains important to guide COVID-19 vaccine composition and recommendations.

## Key Points

**Question:**

What was the estimated vaccine effectiveness (VE) of the 2024-2025 COVID-19 vaccines against severe COVID-19, and did it vary by SARS-CoV-2 lineage or spike protein mutations?

**Findings:**

In this case-control study of 1888 adults with COVID-19 and 6605 adults without COVID-19, estimated VE was 40% against hospitalization and 79% against invasive mechanical ventilation or death. The estimated VE was similar for KP.3.1.1 and XEC lineages, as well as for spike protein mutations potentially associated with immune evasion (S31 deletion, T22N and F59S substitutions).

**Meaning:**

These findings suggest that COVID-19 vaccines offered protection against hospitalization and severe in-hospital outcomes during the 2024-2025 season, in which multiple JN.1 lineages evolved and circulated.

## Teaser

This case-control study estimates vaccine effectiveness of the 2024-2025 COVID-19 vaccines against severe COVID-19 and investigates whether it varied by SARS-CoV-2 lineage or spike protein mutations.

## Introduction

COVID-19 remains a public health threat, with an estimated 380 000 to 540 000 hospitalizations and 44 000 to 63 000 deaths occurring in the US during the 2024-2025 season.^1^ COVID-19 vaccination reduces the likelihood of severe COVID-19,^2^ and timely estimates of vaccine effectiveness (VE) against new SARS-CoV-2 variants may inform decisions on updates to COVID-19 vaccine composition.^3^ In response to the shift in predominance from the XBB to JN.1 lineages in January 2024, the US Food and Drug Administration (FDA) approved updated 2024-2025 Moderna and Pfizer-BioNTech monovalent vaccines based on KP.2 and an updated Novavax vaccine based on JN.1 in August 2024.^4^

During the 2024-2025 COVID-19 season, circulating SARS-CoV-2 lineages remained JN.1 descendants with no major strain replacement event.^5^ Instead, JN.1 descendant lineages repeatedly acquired spike protein substitutions and deletions in the N-terminal and receptor-binding domains that were associated with increased in vitro immune evasion relative to JN.1.^5,6,7,8,9,10,11^ JN.1 descendants KP.3.1.1 and XEC contain 3 to 4 spike protein differences compared with KP.2 and were prevalent in US genomic surveillance in late 2024; LP.8.1 contains 6 spike protein differences and increased starting early 2025.^5,12^ During September 2024 through early June 2025, receipt of a 2024-2025 COVID-19 vaccine was recommended for all persons in the US aged 6 months or older^2,13^; vaccination coverage among adults participating in the National Immunization Survey reached 23% overall and 44% among adults aged 65 years or older.^14^

We estimated the VE of the 2024-2025 COVID-19 vaccines in preventing COVID-19–associated hospitalizations and severe in-hospital outcomes among adults aged 18 years or older in the US. Using whole-genome sequencing, we also characterized lineage- and mutation-specific VE against COVID-19–associated hospitalization.

## Methods

This case-control study was conducted by the Investigating Respiratory Viruses in the Acutely Ill (IVY) Network, a multicenter surveillance network comprising 26 hospitals in 20 US states. This activity was reviewed by the Centers for Disease Control and Prevention and each participating institution in the IVY Network, deemed public health surveillance and not research with waiver of participant informed consent, and was conducted consistent with applicable federal law and Centers for Disease Control and Prevention policy (45 CFR part 46.102[1][2], 21 CFR part 56, 42 USC section 241[d], 5 USC section 552a, 44 USC section 3501 et seq). The study followed the Strengthening the Reporting of Observational Studies in Epidemiology (STROBE) reporting guideline.^15^

The IVY Network uses a test-negative, case-control design to assess VE, and methods have been described previously.^16,17,18,19^ Briefly, site personnel prospectively enrolled adult patients aged 18 years or older admitted to IVY Network hospitals between September 1, 2024, and April 30, 2025, who met a COVID-19–like illness case definition (eMethods in Supplement 1) and underwent SARS-CoV-2 clinical testing. Enrolled patients were tested clinically for SARS-CoV-2 at their local hospital and had nasal swab specimens collected, which were tested at a central laboratory at Vanderbilt University Medical Center for SARS-CoV-2, influenza viruses, respiratory syncytial virus (RSV), and human metapneumovirus using real-time reverse-transcription polymerase chain reaction (eMethods in Supplement 1). The case condition was defined by a positive test result for SARS-CoV-2 in local or central laboratories for a specimen collected within 10 days of symptom onset and 3 days of hospital admission. Case patients with known coinfections (ie, influenza viruses, RSV, human metapneumovirus) were excluded because COVID-19 vaccination would not be expected to prevent hospitalization caused by other respiratory viruses. The control condition was defined by a negative test for SARS-CoV-2. Control patients with positive test results for influenza (all adults) or RSV (adults aged ≥60 years) were excluded due to the correlation between COVID-19 and influenza or RSV vaccination behaviors, which can bias VE estimates.^20,21,22^ Demographic and clinical data were collected through electronic medical record (EMR) review and patient or proxy interview. Self-reported race and ethnicity (Hispanic or Latino of any race, non-Hispanic Black, non-Hispanic White, non-Hispanic other race [American Indian or Alaska Native, Asian, Native Hawaiian or Pacific Islander, or other; combined because of small counts], or unknown) were collected because of their potential association with COVID-19 case and/or vaccination status.

### Classification of Vaccination Status

Verification of COVID-19 vaccination status was performed using hospital EMRs, immunization information systems (IISs) (ie, state or local vaccine registries), or patient or proxy interviews using a hierarchical approach. Evidence of vaccination from either EMRs or IISs was preferentially used, even if interview data were available. If EMR or IIS data were unavailable, plausible interview data with known location and date of COVID-19 vaccine receipt were used.^23^ Patients were classified into 2 vaccination groups: (1) those who received a 2024-2025 COVID-19 vaccine dose (either BNT162b2 [Pfizer-BioNTech], mRNA-1273 [Moderna], or NVX-CoV2705 [Novavax]) at least 7 days before illness onset and (2) those who did not receive a 2024-2025 dose, representing both patients who had received previous COVID-19 vaccine doses and patients who had never received a COVID-19 vaccine dose. We excluded patients if they received a 2024-2025 dose less than 7 days before illness onset or received more than one 2024-2025 dose.

### Sequencing Methods

SARS-CoV-2–positive specimens were sent to the University of Michigan for whole-genome sequencing to identify spike protein mutations and lineages. Sequencing was performed using the Midnight protocol on a GridION instrument (Oxford Nanopore Technologies). Sequences were considered adequate for lineage identification if they had a Nextclade, version 3.4.1 genome coverage of at least 80% and quality-control status of good or mediocre. Lineages were defined from the following clades identified using Nextstrain nomenclature (representative Pango lineage shown in parentheses): 24A (JN.1), 24B (JN.1.11.1), 24C (KP.3), 24D (XDV.1), 24E (KP.3.1.1), 24F (XEC), 24G (KP.2.3), 24H (LF.7), 25A (LP.8.1), and 25C (XFG). Spike amino acid substitutions and deletions were identified and parsed from Nextclade output.

### Immunocompromise Status and Severe In-Hospital Outcomes

Immunocompromising conditions included active solid tumor or hematologic malignant neoplasm (defined as newly diagnosed malignant neoplasm or treatment within the past 6 months), solid organ transplant, hematopoietic cell transplant, HIV infection, congenital immunodeficiency syndrome, use of an immunosuppressive medication within the past 30 days, splenectomy, or another condition that causes moderate or severe immunosuppression. The following severe in-hospital outcomes were characterized from hospital admission to the first of hospital discharge, patient death, or hospital day 28 (eMethods in Supplement 1)^16^: (1) supplemental oxygen therapy (defined as supplemental oxygen at any flow rate and by any device for patients not receiving chronic oxygen therapy or with escalation of oxygen therapy for patients receiving chronic oxygen therapy), (2) acute respiratory failure defined as new receipt of high-flow nasal cannula, noninvasive ventilation, or invasive mechanical ventilation [IMV]), (3) intensive care unit (ICU) admission, and (4) a composite of IMV or death. To test the hypothesis that VE differed by outcome, *P *values and 95% CIs for differences in VE against hospitalization vs VE against severe outcomes were calculated using bootstrapping with 10 000 replicates (eMethods in Supplement 1).

### Statistical Analysis

Descriptive comparisons were made using Pearson χ^2^ test for categorical variables and the Mann-Whitney *U* test for continuous variables. Multivariable logistic regression was used to estimate the odds of 2024-2025 COVID-19 vaccination between case and control patients. Odds ratios (ORs) were adjusted for age (continuous), sex (male, female), race and ethnicity, US Department of Health and Human Services region, admission date in biweekly intervals, and Charlson Comorbidity Index (0, 1-2, 3-4, 5-6, or ≥7). The VE against COVID-19–associated outcomes was calculated as (1 − adjusted OR) × 100%. Estimates of VE were calculated separately for age group, immunocompromise status, time since vaccination strata (either 60- or 90-day windows), severe in-hospital outcomes, and case patients with lineage (Nextstrain clade) or spike protein mutations. Statistical significance was indicated by a 2-sided *P* < .05. Analyses were performed using R, version 4.4.3 (R Foundation for Statistical Computing).

## Results

Among 8493 adults included from 26 hospitals in the IVY Network during the study period, 1888 were designated as case patients with COVID-19 and 6605 as control patients (Table; eFigures 1 and 2 in Supplement 1). Among included patients (median [IQR] age, 66 [54-76] years; 4338 female [51.1%] and 4155 male [48.9%]; 1061 identifying as Hispanic or Latino ethnicity [12.5%], 1905 as non-Hispanic Black race [22.4%], 4883 as non-Hispanic White race [57.5%], 434 as non-Hispanic other race [5.1%], and 210 as unknown race and ethnicity [2.5%]), the median Charlson Comorbidity Index was 4 (IQR, 3-6). Control patients were younger than case patients (median [IQR], 65 years [53-75] vs 71 [59-80 years] years; *P* < .001) and had a lower Charlson Comorbidity Index (median [IQR], 4 [2-6] vs 5 [3-7]; *P* < .001). Distributions of sex, race and ethnicity, and enrollment site were similar.

**Table.** Characteristics of Hospitalized Adults Aged 18 Years or Older With COVID-19–Like Illness by COVID-19 Case-Control and Vaccination Status

Characteristic	Patients, No. (%)^a^					
	Overall (N = 8493)	COVID-19 case-control status			2024-2025 COVID-19 vaccination status	
		Case patients with COVID-19 (n = 1888)	Case patients with COVID-19 and sequenced specimens (n = 951)	Control patients with negative test results (n = 6605)	Received vaccine (n = 1440)	Did not receive vaccine (n = 7053)
Received 2024-2025 COVID-19 vaccine^b^	1440 (17.0)	216 (11.4)	105 (11.0)	1224 (18.5)	1440 (100)	NA
Age, median (IQR), y	66 (54-76)	71 (59-80)	73 (62-82)	65 (53-75)	72 (63-80)	65 (53-75)
Age group, y						
18-64	3844 (45.3)	666 (35.3)	280 (29.4)	3178 (48.1)	410 (28.5)	3434 (48.7)
≥65	4649 (54.7)	1222 (64.7)	671 (70.6)	3427 (51.9)	1030 (71.5)	3619 (51.3)
Sex						
Female	4338 (51.1)	966 (51.2)	493 (51.8)	3372 (51.1)	697 (48.4)	3641 (51.6)
Male	4155 (48.9)	922 (48.8)	458 (48.2)	3233 (48.9)	743 (51.6)	3412 (48.4)
Race and ethnicity						
Hispanic or Latino of any race	1061 (12.5)	229 (12.1)	122 (12.8)	832 (12.6)	117 (8.1)	944 (13.4)
Non-Hispanic Black	1905 (22.4)	377 (20.0)	165 (17.4)	1528 (23.1)	237 (16.5)	1668 (23.6)
Non-Hispanic White	4883 (57.5)	1132 (60.0)	582 (61.2)	3751 (56.8)	986 (68.5)	3897 (55.3)
Non-Hispanic other race^c^	434 (5.1)	98 (5.2)	57 (6.0)	336 (5.1)	80 (5.6)	354 (5.0)
Unknown	210 (2.5)	52 (2.8)	25 (2.6)	158 (2.4)	20 (1.4)	190 (2.7)
DHHS region (headquarters)^d^						
1 (Boston)	1708 (20.1)	477 (25.3)	279 (29.3)	1231 (18.6)	323 (22.4)	1385 (19.6)
2 (New York)	499 (5.9)	99 (5.2)	63 (6.6)	400 (6.1)	22 (1.5)	477 (6.8)
3 (Philadelphia)	216 (2.5)	54 (2.9)	21 (2.2)	162 (2.5)	53 (3.7)	163 (2.3)
4 (Atlanta)	1226 (14.4)	249 (13.2)	125 (13.1)	977 (14.8)	79 (5.5)	1147 (16.3)
5 (Chicago)	1655 (19.5)	365 (19.3)	122 (12.8)	1290 (19.5)	339 (23.5)	1316 (18.7)
6 (Dallas)	658 (7.7)	151 (8.0)	71 (7.5)	507 (7.7)	84 (5.8)	574 (8.1)
7 (Kansas City)	293 (3.4)	48 (2.5)	25 (2.6)	245 (3.7)	41 (2.8)	252 (3.6)
8 (Denver)	1089 (12.8)	207 (11.0)	111 (11.7)	882 (13.4)	274 (19.0)	815 (11.6)
9 (San Francisco)	778 (9.2)	168 (8.9)	104 (10.9)	610 (9.2)	141 (9.8)	637 (9.0)
10 (Seattle)	371 (4.4)	70 (3.7)	30 (3.2)	301 (4.6)	84 (5.8)	287 (4.1)
Immunocompromised	2362 (27.8)	453 (24.0)	223 (23.4)	1909 (28.9)	437 (30.3)	1925 (27.3)
Charlson Comorbidity Index, median (IQR)	4 (3-6)	5 (3-7)	5 (3-7)	4 (2-6)	5 (3-7)	4 (2-6)
Time of COVID-19–like illness hospitalization						
September 2024	1224 (14.4)	481 (25.5)	240 (25.2)	743 (11.2)	18 (1.3)	1206 (17.1)
October 2024	947 (11.2)	292 (15.5)	154 (16.2)	655 (9.9)	83 (5.8)	864 (12.3)
November 2024	821 (9.7)	232 (12.3)	117 (12.3)	589 (8.9)	108 (7.5)	713 (10.1)
December 2024	1011 (11.9)	237 (12.6)	128 (13.5)	774 (11.7)	205 (14.2)	806 (11.4)
January 2025	1170 (13.8)	196 (10.4)	98 (10.3)	974 (14.7)	257 (17.8)	913 (12.9)
February 2025	1071 (12.6)	151 (8.0)	64 (6.7)	920 (13.9)	245 (17.0)	826 (11.7)
March 2025	1160 (13.7)	183 (9.7)	80 (8.4)	977 (14.8)	281 (19.5)	879 (12.5)
April 2025	1089 (12.8)	116 (6.1)	70 (7.4)	973 (14.7)	243 (16.9)	846 (12.0)
In-hospital outcomes among case patients with COVID-19						
Supplemental oxygen therapy	1077 (12.7)	1077 (57.0)	544 (57.2)	NA	114 (7.9)	963 (13.7)
Acute respiratory failure	361 (4.3)	361 (19.1)	162 (17.0)	NA	33 (2.3)	328 (4.7)
ICU admission	333 (3.9)	333 (17.6)	140 (14.7)	NA	27 (1.9)	306 (4.3)
IMV or death	162 (1.9)	162 (8.6)	62 (6.5)	NA	10 (1.0)	152 (2.2)

Abbreviations: DHHS, US Department of Health and Human Services; ICU, intensive care unit; IMV, invasive mechanical ventilation; IVY, Investigating Respiratory Viruses in the Acutely Ill; NA, not applicable.

^a^ Percentages are by column.

^b^ Patients were classified into 2 vaccination groups: (1) those who received a 2024-2025 COVID-19 vaccine dose (either BNT162b2, mRNA-1273, or NVX-CoV2705) at least 7 days before illness onset and (2) those who did not receive a 2024-2025 dose, representing both patients who had received previous monovalent and/or bivalent COVID-19 vaccine doses and patients who had never received a COVID-19 vaccine dose.

^c^ Includes American Indian or Alaska Native, Asian, Native Hawaiian or Pacific Islander, or other.

^d^ The IVY Network hospitals by DHHS region are listed in the eMethods in Supplement 1.

A total of 216 of the 1888 case patients (11.4%) and 1224 of the 6605 control patients (18.5%) received a 2024-2025 COVID-19 vaccine (Table). The prevalence of 2024-2025 COVID-19 vaccination among control patients increased from 2.2% (95% CI, 1.3%-3.5%) in September 2024 to 14.8% (95% CI, 12.1%-17.9%) in November 2024 before plateauing at 22.7% (95% CI, 20.2%-25.4%) in January 2025 (eFigure 3 in Supplement 1). Documentation of vaccination status was based on hospital EMR or vaccine registries, where available (1274 of 1440 patients [88.5%]), and plausible patient or proxy interviews otherwise (166 of 1440 patients [11.5%]). Among 1251 patients with known product type information, 793 (63.4%) received BNT162b2, 428 (34.2%) received mRNA-1273, and 30 (2.4%) received NVX-CoV2705.

### COVID-19 VE Against Hospitalization and Severe In-Hospital Outcomes

**Figure 1.**
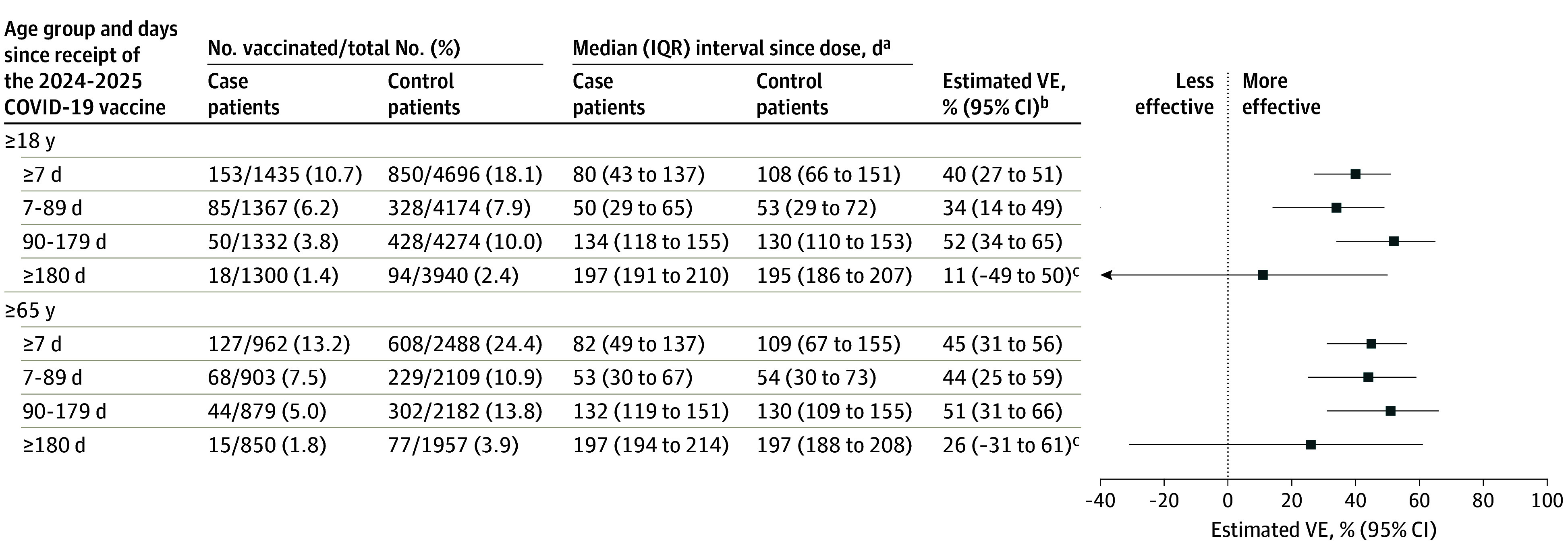
Vaccine Effectiveness (VE) of 2024-2025 COVID-19 Vaccines Against COVID-19–Associated Hospitalization Among Immunocompetent Adults by Time Since Dose Receipt and Age Group ^a^Time since vaccination with a 2024-2025 COVID-19 vaccine. ^b^The VE was calculated by comparing the odds of 2024-2025 COVID-19 vaccination in case and control patients, as detailed in the Methods. ^c^Estimates are imprecise due to limited numbers of enrolled patients with dose receipt of at least 180 days earlier than hospitalization. This imprecision indicates that the actual VE may be substantially different from the point estimate shown, and estimates should therefore be interpreted with caution.

Among 6131 immunocompetent adults aged 18 years or older, overall effectiveness of the 2024-2025 COVID-19 vaccines against COVID-19–associated hospitalization was 40% (95% CI, 27%-51%), with a median time since dose receipt of 80 days (IQR, 43-137 days) among case patients and 108 days (IQR, 66-151 days) among control patients. The VE was 34% (95% CI, 14%-49%) 7 to 89 days after vaccination and 52% (95% CI, 34%-65%) 90 to 179 days after vaccination (Figure 1), with similar trends by 60-day strata (eFigure 4 in Supplement 1). Among 3450 immunocompetent adults aged 65 years or older, VE was 45% (95% CI, 31%-56%) overall, 44% (95% CI, 25%-59%) 7 to 89 days after vaccination, and 51% (95% CI, 31%-66%) 90 to 179 days after vaccination. Overall VE for immunocompromised adults aged 65 years or older (n = 1199) was 36% (95% CI, 6%-57%), with a median time since dose receipt of 79 days (IQR, 49-131 days) among case patients and 106 days (IQR, 56-153 days) among control patients (eFigure 5 in Supplement 1).

**Figure 2.**
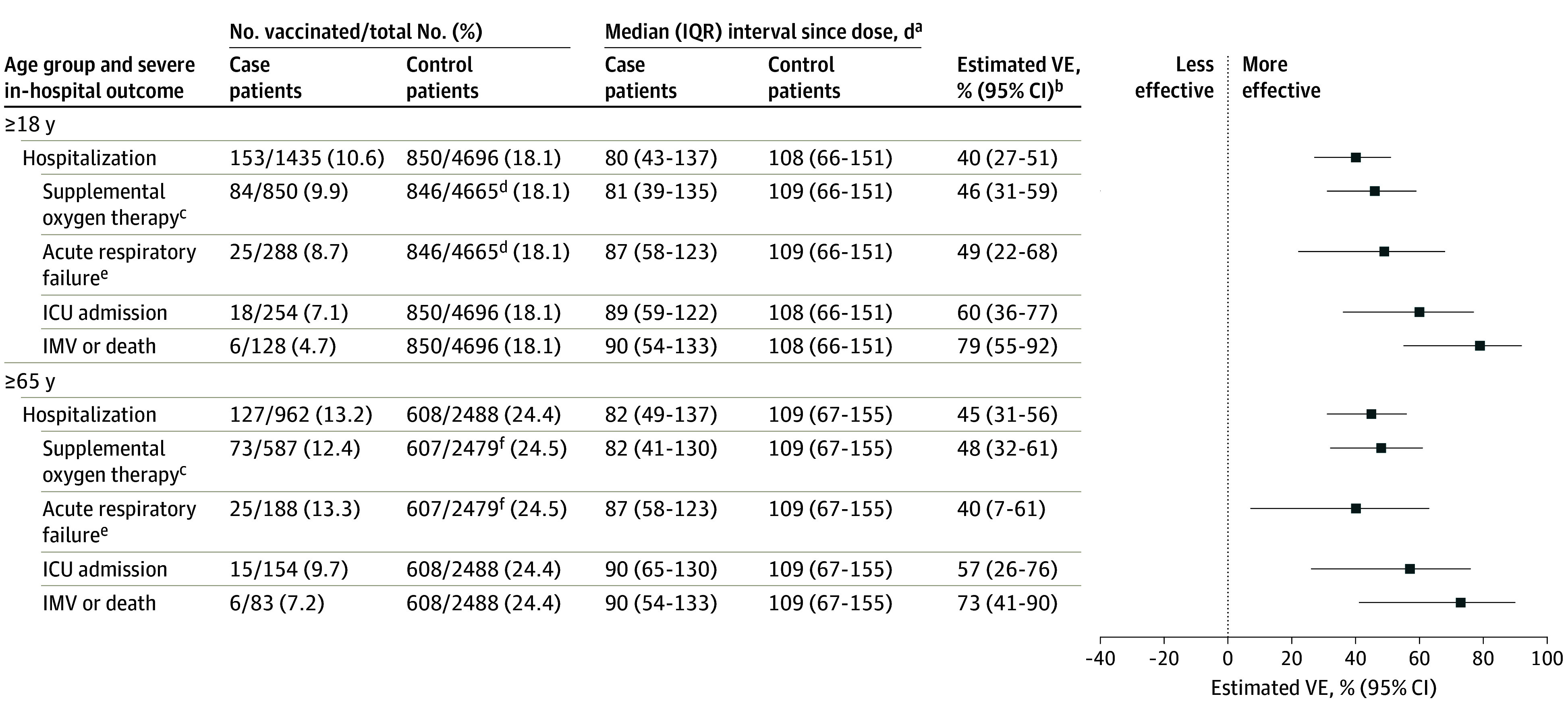
Vaccine Effectiveness (VE) of 2024-2025 COVID-19 Vaccines Against COVID-19–Associated Severe In-Hospital Outcomes Among Immunocompetent Adults by Age Group and Outcome ICU indicates intensive care unit; IMV, invasive mechanical ventilation. ^a^Time since vaccination with a 2024-2025 COVID-19 vaccine. ^b^The VE was calculated by comparing the odds of 2024-2025 COVID-19 vaccination in case and control patients, as detailed in the Methods. ^c^Defined as supplemental oxygen at any flow rate and by any device for those not on chronic oxygen therapy or with escalation of oxygen therapy for patients receiving chronic oxygen therapy. ^d^This denominator is lower because patients receiving home IMV prior to the acute illness were not eligible for this outcome (n = 31). ^e^Defined as new receipt of high-flow nasal cannula, noninvasive ventilation, or IMV. ^f^This denominator is lower because patients receiving home IMV prior to the acute illness were not eligible for this outcome (n = 9).

Among 1888 case patients, 1077 (57.0%) received supplemental oxygen therapy, 361 (19.1%) experienced acute respiratory failure, 333 (17.6%) were admitted to the ICU, and 162 (8.6%) received IMV or died (Table). The VE of a 2024-2025 dose for immunocompetent adults was 46% (95% CI, 31%-59%) against supplemental oxygen therapy, 49% (95% CI, 22%-68%) against acute respiratory failure, 60% (95% CI, 36%-77%) against ICU admission, and 79% (95% CI, 55%-92%) against receipt of IMV or death (Figure 2). Point estimates increased with more severe outcomes, and VE against IMV or death was significantly higher than VE against hospitalization (difference in VE estimates, 39% [bootstrap 95% CI, 18%-57%]; bootstrap *P* = .004) (eTable 1 in Supplement 1). Estimates were similar for immunocompetent adults aged 65 years or older, though VE was not significantly different between in-hospital outcomes and hospitalization (Figure 2; eTable 1 in Supplement 1).

### COVID-19 VE by SARS-CoV-2 Lineage and Spike Protein Mutations

**Figure 3.**
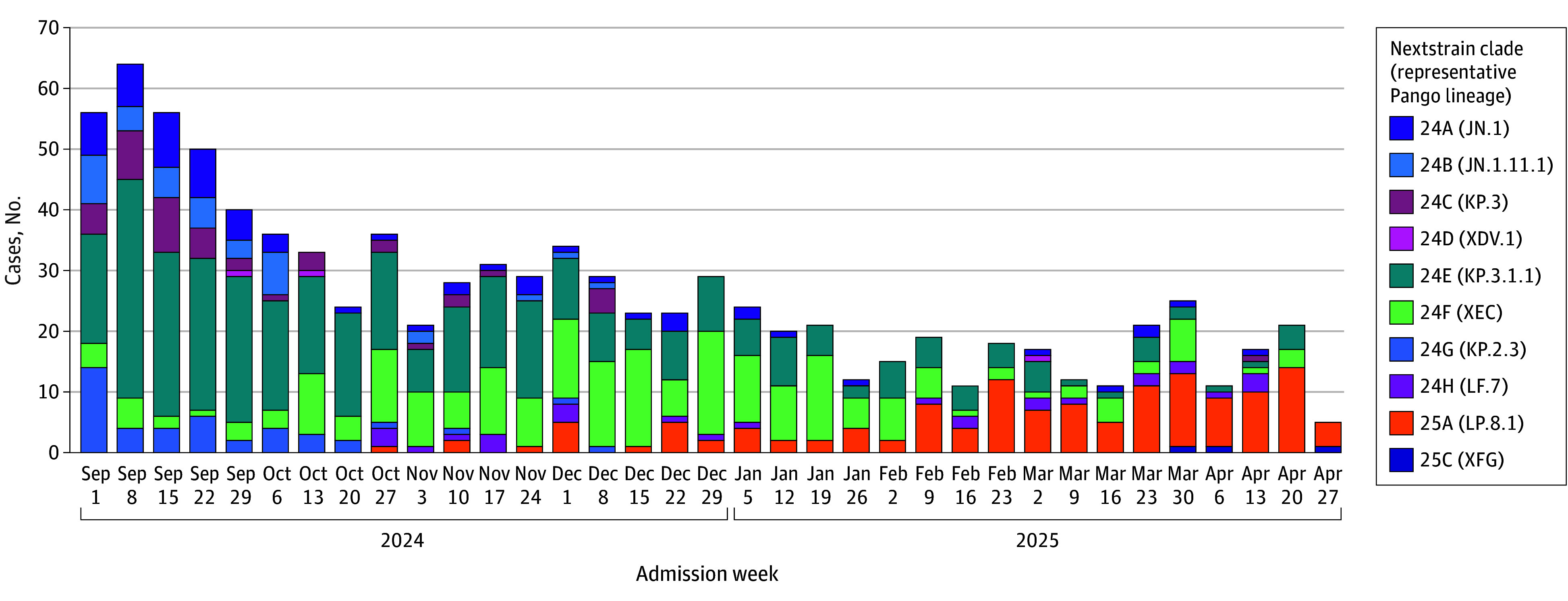
Number of COVID-19 Cases by Hospital Admission Week and SARS-CoV-2 Lineage Dates are for the start of the admission week. SARS-CoV-2 lineage was identified using Nextstrain after conducting viral whole-genome sequencing.

Identification of SARS-CoV-2 lineage through whole-genome sequencing was successful for 951 case patients (50.4%). Case patients with sequencing data had similar distributions of clinical and demographic characteristics compared with all case patients with COVID-19 (Table). In a sensitivity analysis, VE by time since dose was similar for case patients with sequencing data compared with all case patients with COVID-19 (eFigure 6 in Supplement 1). Among case patients with sequencing data, 348 (36.6%) had KP.3.1.1 lineage infection, and 218 (22.9%) had XEC, 134 (14.1%) had LP.8.1, and 251 (26.4%) had other JN.1 descendant lineages (Figure 3; eTable 2 in Supplement 1). KP.3.1.1 was most prevalent in September and October 2024, after which prevalence of XEC increased steadily from November 2024 to January 2025 before being displaced by LP.8.1 by February 2025. Nearly all circulating lineages contained either the S31 deletion (n = 602 [63.3%]), T22N substitution (n = 290 [30.5%]), or F59S substitution (n = 243 [25.6%]) in the N-terminal domain of the spike protein (eFigure 7 in Supplement 1). The S31 deletion was more common among KP.3.1.1 (347 of 348 [99.7%]), KP.2.3 (43 of 43 [100%]), and LP.8.1 (134 of 134 [100%]), and the T22N substitution was more common among XEC (217 of 218 [99.5%]) and LF.7 (28 of 28 [100%]) (eTable 3 in Supplement 1).

**Figure 4.**
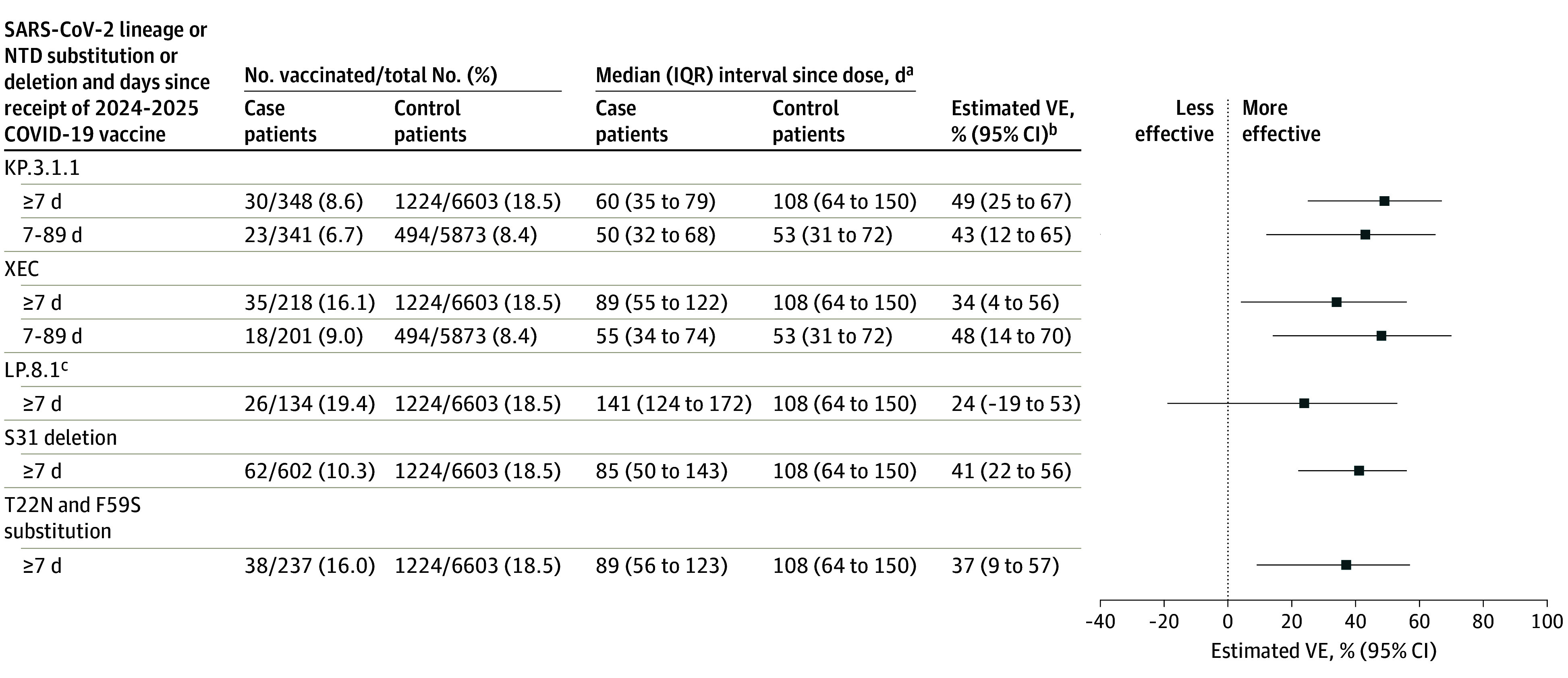
Vaccine Effectiveness (VE) of 2024-2025 COVID-19 Vaccines Against Hospitalization Among Adults Aged 18 Years or Older by SARS-CoV-2 Lineage and N-Terminal Domain (NTD) Substitutions or Deletions SARS-CoV-2 lineage was identified using Nextstrain after conducting viral whole-genome sequencing. ^a^Time since vaccination with a 2024-2025 COVID-19 vaccine. ^b^The VE was calculated by comparing the odds of 2024-2025 COVID-19 vaccination in case and control patients, as detailed in the Methods. Some estimates are imprecise, which might be due to a relatively small number of patients in each level of vaccination or case status. This imprecision indicates that the actual VE may be substantially different from the point estimate shown, and estimates should therefore be interpreted with caution. ^c^Limited numbers of patients with LP.8.1 infection were 7 to 89 days from their updated dose, precluding estimation of VE within this stratum.

A total of 30 case patients (8.6%) with KP.3.1.1, 35 (16.1%) with XEC, and 26 (19.4%) with LP.8.1 lineage infections received a 2024-2025 COVID-19 vaccine dose. Among adults aged 18 years or older with available sequencing data (including both immunocompetent and immunocompromised patients), VE was 49% (95% CI, 25%-67%) against KP.3.1.1-associated hospitalization, 34% (95% CI, 4%-56%) against XEC-associated hospitalization, and 24% (95% CI, −19% to 53%) against LP.8.1-associated hospitalization (Figure 4). Due to sequential circulation (Figure 3), median time since dose receipt among case patients was highest for LP.8.1 (141 days [IQR, 124-172 days]) compared with XEC and KP.3.1.1 (*P* < .001) and higher for XEC (89 days [IQR, 55-122 days]) compared with KP.3.1.1 (60 days [IQR, 35-79 days]) (*P* = .04). Restricting to the first 7 to 89 days following vaccination, VE was 43% (95% CI, 12%-65%) against KP.3.1.1-associated hospitalization and 48% (95% CI, 14%-70%) against XEC-associated hospitalization (Figure 4). The VE was 41% (95% CI, 22%-56%) against SARS-CoV-2 strains with the S31 deletion and 37% (95% CI, 9%-57%) against strains with the T22N and F59S substitutions, with similar time since dose receipt among case patients (85 [IQR, 50-143] and 89 [IQR, 56-123] days, respectively) (Figure 4).

## Discussion

This case-control study within a multicenter US surveillance network found that 2024-2025 COVID-19 vaccines were 40% effective against COVID-19–associated hospitalizations among immunocompetent adults enrolled September 2024 to April 2025. Protection was sustained until at least 3 to 6 months following vaccination. Vaccine effectiveness was highest against the most severe outcomes of IMV or death (79%), consistent with earlier reports during the COVID-19 pandemic.^17,24,25,26,27,28,29^ Vaccine effectiveness among all adults was similar against KP.3.1.1-associated and XEC-associated hospitalizations in the first 3 months after vaccination (43% and 48%, respectively). It was lower against LP.8.1-associated hospitalization (24%), with 95% CIs overlapping the null, but estimates were imprecise, and time since vaccination was longer compared with other lineages. Vaccine effectiveness was similar against variants with spike S31 deletions or T22N and F59S substitutions (41% and 37%, respectively). Taken together, these findings suggest that JN.1- and KP.2-based COVID-19 vaccines were effective against multiple JN.1 descendent lineages that emerged and cocirculated during the 2024-2025 season.

Our findings that 2024-2025 COVID-19 vaccines were associated with added protection against hospitalization are concordant with other US estimates, including 2 EMR-based networks reporting a VE of 68% and 45%, with a median of 30 and 53 days following vaccination, respectively.^30,31^ Our estimates are also generally consistent with VE of JN.1-based vaccines from the UK (43% 10-14 weeks after vaccination),^32^ Denmark (70% against hospitalization and 76% against death for BNT162b2 vaccination; time since vaccination not reported),^33^ and a multinational outpatient European network (66% against medically attended illness a median of 41 days after vaccination).^34^ We observed a lower VE point estimate 7 to 89 days after vaccination compared with 90 to 179 days after vaccination, similar to findings from the UK.^32^ This lower VE point estimate may have been influenced by transient increases in population immunity from increased SARS-CoV-2 circulation in late summer 2024 just prior to availability of the 2024-2025 COVID-19 vaccines. Increases in population immunity can result in lower estimates of COVID-19 VE because VE is a measure of incremental protection beyond prior vaccination or infection.^35,36^ Few studies have evaluated protection against in-hospital outcomes this season, and our finding of higher VE against COVID-19–associated IMV or death shows the importance of recent vaccination to prevent the most severe outcomes following infection.

Few observational studies have evaluated lineage-specific VE during the 2024-2025 season. One challenge is that several JN.1 descendants cocirculated without clearly defined periods of predominance (Figure 3). In such a scenario, lineage-specific VE estimates based on calendar time to delineate predominance periods could be biased due to lineage misclassification error,^37^ and whole-genome sequencing of patient specimens may be a more reliable approach for estimation. A study combining whole-genome sequencing and national registry data in Denmark found similar protection of JN.1-based vaccines against hospitalization with KP.3.1.1, XEC, and any variant.^33^ Immunogenicity studies have also found strong increases in neutralizing antibody responses to KP.3.1.1 and XEC following JN.1- and KP.2-based booster administration.^38,39,40^ We were unable to distinguish natural waning from variant-mediated immune escape for LP.8.1 due to limited sample sizes, but antigenic cartography data have suggested that LP.8.1 is antigenically similar to other JN.1 descendants,^41,42^ and JN.1- and KP.2-based vaccination yielded similar antibody titers against LP.8.1, KP.3.1.1, and XEC in some cohorts.^42,43,44,45^

N-terminal domain mutations, including the S31 deletion (present in KP.3.1.1, LP.8.1, and other lineages) and T22N and F59S substitutions (present in XEC), are a novel occurrence for SARS-CoV-2 and may confer fitness advantages through introduction of glycosylation sites, which can disrupt N-terminal domain-binding antibodies.^6,7,9,10,11^ The S31 deletion and F59S substitution may also induce conformational changes in SARS-CoV-2 spike protein that could affect antibody binding to the receptor-binding domain.^10^ Here, we found that these N-terminal mutations were prevalent among patients hospitalized with COVID-19 in the US and that the 2024-2025 COVID-19 vaccines provided similar protection against lineages with S31 deletion vs T22N and F59S substitutions.^9^

### Limitations

This analysis had several limitations. First, the sequential timing of KP.3.1.1, XEC, and LP.8.1 circulation correlated with increasing time since vaccination, which made it challenging to differentiate natural immunologic waning from variant immune evasion, particularly for LP.8.1. Therefore, it is unclear whether the lower point estimate for LP.8.1 was due to genetic changes or increased time since dose. Second, decreasing counts of COVID-19 hospitalizations in spring 2025 precluded precise estimation of VE beyond 180 days after vaccine receipt. Third, this analysis did not adjust for the number or timing of prior SARS-CoV-2 infections or vaccinations, which can influence estimates of VE.^35^ Fourth, while we attempted to sequence specimens from all case patients, we were unable to identify lineage in approximately half potentially due to low viral load or viral degradation. Exclusion of these patients did not seem to substantially bias VE (eFigure 5 in Supplement 1).^46^ Fifth, although IVY is a multicenter network with hospitals in geographically diverse locations, our results may not be generalizable to the entire US. Finally, although analyses were adjusted for some relevant confounders, residual confounding may remain.

## Conclusions

This multicenter, case-control study found that the VE of 2024-2025 COVID-19 vaccines was associated with protection against COVID-19 hospitalization and severe in-hospital outcomes and against multiple JN.1 descendants. During a season without major antigenic changes to circulating SARS-CoV-2 viruses, we found sustained protection from COVID-19 vaccines through at least 90 to 179 days after vaccination. In June 2025, the Food and Drug Administration recommended monovalent JN.1 lineage–based COVID-19 vaccines, preferentially using the LP.8.1 strain, for 2025-2026 COVID-19 vaccine formulations.^47^ Monitoring COVID-19 VE, including stratifying by SARS-CoV-2 lineage and spike protein mutations, remains important to guide COVID-19 vaccine composition and recommendations.^3^

## Notes

Supplement 1.
eMethods.

eReferences.
**eTable 1.** Bootstrap-Based P Values and 95% CIs for Differences in the Effectiveness of the 2024-2025 COVID-19 Vaccine Against COVID-19–Associated Hospitalization vs In-Hospital Severe Outcomes **eTable 2.** Number of COVID-19 Cases by Hospital Admission Week and SARS-COV-2 Lineage **eTable 3.** Counts of Spike Protein N-Terminal Domain Substitutions or Deletion by Nextstrain Clade **eFigure 1.** Participant Flow Diagram and Analytic Cohorts **eFigure 2.** Numbers of COVID-19 Case and Test-Negative Control Participants by Admission Week **eFigure 3.** 2024-2025 COVID-19 Vaccination Coverage by Admission Week Among Test-Negative Control Participants **eFigure 4.** Effectiveness of 2024-2025 COVID-19 Vaccine Against COVID-19–Associated Hospitalization Among Immunocompetent Adults by Time Since Dose Receipt (60-Day Windows) and Age Group **eFigure 5.** Effectiveness of 2024-2025 COVID-19 Vaccine Against COVID-19–Associated Hospitalization Among Immunocompromised Adults by Age Group **eFigure 6.** Effectiveness of 2024-2025 COVID-19 Vaccine Against COVID-19–Associated Hospitalization Among Case Participants With SARS-CoV-2 Lineage Successfully Identified Through Whole-Genome Sequencing and Among All Case Participants by Time Since Dose Receipt **eFigure 7.** Number of COVID-19 Case by Hospital Admission Week and SARS-CoV-2 Spike Protein N-Terminal Domain Substitutions and Deletions

Supplement 2.
Nonauthor Collaborators

Supplement 3.
Data Sharing Statement
