# Supplementary material for: Estimated Effectiveness of 2024-2025 COVID-19 Vaccination Against Severe COVID-19
Source: JAMA Netw Open. 2026 Feb 3;9(2):e2557415. doi: 10.1001/jamanetworkopen.2025.57415 (PMC12869339; doi:10.1001/jamanetworkopen.2025.57415)
Supplement: Supplement 1. — eMethods. eReferences. eTable 1. Bootstrap-Based P Values and 95% CIs for Differences in the Effectiveness of the 2024-2025 COVID-19 Vaccine Against COVID-19–Associated Hospitalization vs In-Hospital Severe Outcomes eTable 2. Number of COVID-19 Cases by Hospital Admission Week and SARS-COV-2 Lineage eTable 3. Counts of Spike Protein N-Terminal Domain Substitutions or Deletion by Nextstrain Clade eFigure 1. Participant Flow Diagram and Analytic Cohorts eFigure 2. Numbers of COVID-19 Case and Test-Negative Control Participants by Admission Week eFigure 3. 2024-2025 COVID-19 Vaccination Coverage by Admission Week Among Test-Negative Control Participants eFigure 4. Effectiveness of 2024-2025 COVID-19 Vaccine Against COVID-19–Associated Hospitalization Among Immunocompetent Adults by Time Since Dose Receipt (60-Day Windows) and Age Group eFigure 5. Effectiveness of 2024-2025 COVID-19 Vaccine Against COVID-19–Associated Hospitalization Among Immunocompromised Adults by Age Group eFigure 6. Effectiveness of 2024-2025 COVID-19 Vaccine Against COVID-19–Associated Hospitalization Among Case Participants With SARS-CoV-2 Lineage Successfully Identified Through Whole-Genome Sequencing and Among All Case Participants by Time Since Dose Receipt eFigure 7. Number of COVID-19 Case by Hospital Admission Week and SARS-CoV-2 Spike Protein N-Terminal Domain Substitutions and Deletions [file jamanetwopen-e2557415-s001.pdf]

Supplemental Online Content

Ma KC, Webber A, Lauring AS, et al; Investigating Respiratory Viruses in the Acutely Ill (IVY) Network. Estimated effectiveness of 2024-2025 COVID-19 vaccination against severe COVID-19. *JAMA Netw Open*. 2026;9(2):e2557415. doi:10.1001/jamanetworkopen.2025.57415

eMethods.

eReferences.

eTable 1. Bootstrap-Based *P* Values and 95% CIs for Differences in the Effectiveness of the 2024-2025 COVID-19 Vaccine Against COVID-19–Associated Hospitalization vs In-Hospital Severe Outcomes

eTable 2. Number of COVID-19 Cases by Hospital Admission Week and SARS-COV-2 Lineage

eTable 3. Counts of Spike Protein N-Terminal Domain Substitutions or Deletion by Nextstrain Clade

eFigure 1. Participant Flow Diagram and Analytic Cohorts

eFigure 2. Numbers of COVID-19 Case and Test-Negative Control Participants by Admission Week

eFigure 3. 2024-2025 COVID-19 Vaccination Coverage by Admission Week Among Test-Negative Control Participants

eFigure 4. Effectiveness of 2024-2025 COVID-19 Vaccine Against COVID-19–Associated Hospitalization Among Immunocompetent Adults by Time Since Dose Receipt (60-Day Windows) and Age Group

eFigure 5. Effectiveness of 2024-2025 COVID-19 Vaccine Against COVID-19–Associated Hospitalization Among Immunocompromised Adults by Age Group

eFigure 6. Effectiveness of 2024-2025 COVID-19 Vaccine Against COVID-19–Associated Hospitalization Among Case Participants With SARS-CoV-2 Lineage Successfully Identified Through Whole-Genome Sequencing and Among All Case Participants by Time Since Dose Receipt

eFigure 7. Number of COVID-19 Case by Hospital Admission Week and SARS-CoV-2 Spike Protein N-Terminal Domain Substitutions and Deletions

This supplemental material has been provided by the authors to give readers additional information about their work.

© 2026 Ma KC et al. *JAMA Network Open*.

eMethods

1. COVID-19 case definition

COVID-19–like illness was defined as one or more of the following: fever, cough, shortness of breath, new or worsening findings on chest imaging consistent with pneumonia, or hypoxemia (defined as oxygen saturation <92% or supplemental oxygen use for patients without chronic oxygen needs, or escalation of oxygen therapy for patients on chronic supplemental oxygen).

2. Severe in-hospital outcomes

Clinical severity of COVID-19 cases was characterized using the following severe in-hospital outcomes occurring from hospital presentation to hospital discharge, patient death, or hospital day 28:

i) COVID-19-associated supplemental oxygen therapy
ii) COVID-19-associated acute respiratory failure
iii) COVID-19-associated intensive care unit (ICU) admission
iv) COVID-19-associated invasive mechanical ventilation (IMV) or death

<u>i) COVID-19-associated supplemental oxygen therapy</u>

Patients who met the definition of COVID-19-associated supplemental oxygen therapy either required supplemental oxygen therapy at any time during the hospitalization through day 28 for those not on chronic oxygen or, for patients on chronic supplemental oxygen (**Table**), required an escalation in respiratory support. Supplemental oxygen therapy could be delivered at any flow rate and by any device; this included standard flow oxygen (flow rate <30 liters/minute), high-flow nasal cannula (HFNC), non-invasive ventilation (NIV), and IMV. Patients on home IMV prior to the acute illness were not eligible for this outcome.

Classification of in-hospital respiratory outcome based on type of oxygen or respiratory support used chronically (before illness onset) and highest level received through hospital day 28		
		**Is the patient eligible for this outcome?**

© 2026 Ma KC et al. *JAMA Network Open*.

Chronic pre-illness oxygen use	Oxygen use during hospital course (highest support)	Supplemental oxygen therapy	Acute respiratory failure	Invasive mechanical ventilation
No oxygen use	Standard flow oxygen	Yes	No	No
	High-flow nasal cannula (HFNC)	Yes	Yes	No
	NIV	Yes	Yes	No
	IMV	Yes	Yes	Yes
Standard flow oxygen	Standard flow oxygen	No	No	No
	HFNC	Yes	Yes	No
	NIV	Yes	Yes	No
	IMV	Yes	Yes	Yes
Non-invasive mechanical ventilation (NIV)	Standard flow oxygen	No	No	No
	HFNC	No	No	No
	NIV	No	No	No
	IMV	Yes	Yes	Yes
Invasive mechanical ventilation (IMV)	Standard flow oxygen	No	No	No
	HFNC	No	No	No
	NIV	No	No	No
	IMV	No	No	No

ii) COVID-19-associated acute respiratory failure

Patients were classified as having COVID-19-associated acute respiratory failure treated with advanced respiratory support if they received any of the following during the hospitalization through day 28: HFNC, NIV, or IMV. HFNC was defined as a supplemental oxygen flow rate ≥30 liters per minute. NIV included both continuous positive airway pressure (CPAP) and bilevel positive airway pressure (BiPAP) delivered through a mask. Patients were classified as having NIV use if NIV was received for therapy of the acute illness and not only for treatment of sleep apnea. IMV was defined as positive pressure administered through an endotracheal tube or tracheostomy tube. Patients on home NIV before the acute illness met criteria for this outcome if they had escalation of respiratory support to IMV in the hospital. Patients on home IMV prior to the acute illness were not eligible for this outcome.

© 2026 Ma KC et al. *JAMA Network Open*.

iii) COVID-19-associated intensive care unit (ICU) admission

Patients were classified as having COVID-19-associated ICU admission if they received care in an ICU for any duration of time during the hospitalization through day 28.

iv) COVID-19-associated IMV or death

Patients were classified as having COVID-19-associated IMV or death if they received IMV or died during the hospitalization through day 28. IMV was defined as positive pressure administered through an endotracheal tube or tracheostomy tube. Patients on home IMV prior to the acute illness could not meet the COVID-19-associated IMV or death outcome through receipt of in-hospital IMV.

2. Laboratory Testing Methods

At the time of participant enrollment, a nasal swab specimen was obtained via a fresh swabbing procedure or collection of a residual aliquot in the clinical laboratory. Specimens were stored at -80°C at the enrolling site and shipped to Vanderbilt University Medical Center. Real-time reverse transcription polymerase chain reaction (RT-PCR) testing for SARS-CoV-2, influenza, and RSV was completed at Vanderbilt. Specimens that tested positive for SARS-CoV-2 were submitted to the University of Michigan for viral whole-genome sequencing.

SARS-CoV-2 detection by RT-PCR
Total nucleic acid extract from 100 microliters of upper respiratory specimen collected in viral transport medium was prepared using the MagNA Pure LC Total Nucleic Acid Isolation Kit (Roche Molecular Systems, Pleasanton, CA) and MagNA Pure 96 automated extraction platform (Roche) or QIAamp 96 Virus QiaCube HT kit (Qiagen, Germantown, MD) and QiaCube HT automated extraction system (Qiagen). Extracts (100 microliters eluate volume) were tested by RT-PCR using the QuantStudio 3, QuantStudio 5, or QuantStudio 6 Real-Time PCR System (Applied Biosystems, Waltham, MA) for SARS-CoV-2 nucleocapsid (N)-gene N1 and N2 targets and for the human RNase P (RNP) gene with TaqPath 1-Step RT-qPCR Master Mix CG (Applied Biosystems) according to the *CDC 2019-Novel Coronavirus (2019-nCoV) Real-Time RT-PCR Diagnostic Panel* protocol (https://www.fda.gov/media/134922/download). Qualitative result assignment of *positive*, *not detected*, *inconclusive*, or *invalid specimen* for SARS-CoV-2 RNA was based on the pattern of N1, N2, and RNP Ct values using interpretive criteria delineated in the assay protocol.

Influenza detection by RT-PCR
Total nucleic acid extract from 100 microliters of upper respiratory specimen collected in viral transport medium was prepared using the MagNA Pure LC Total Nucleic Acid Isolation Kit and MagNA Pure 96 automated extraction platform or QIAamp 96 Virus QiaCube HT kit and QiaCube HT automated extraction system. Extracts (100 microliters eluate volume) were tested by RT-PCR using the QuantStudio 3, QuantStudio 5, or QuantStudio 6 Real-Time PCR System

© 2026 Ma KC et al. *JAMA Network Open*.

for influenza A and B using the *CDC Human Influenza Virus Real-Time RT-PCR Diagnostic Panel, Influenza A/B Typing Kit (VER 2)* with Superscript III Platinum One-Step Quantitative RT-PCR System containing ROX passive reference dye (Invitrogen, Waltham, MA). Subtyping of influenza A-positive specimens by RT-PCR was performed using the *CDC Human Influenza Virus Real-Time RT-PCR Diagnostic Panel, Influenza A Subtyping Kit (VER 3)* with Superscript III Platinum One-Step Quantitative RT-PCR System containing ROX passive reference dye. Each specimen also was tested for RNP using TaqPath 1-Step RT-qPCR Master Mix CG (https://www.fda.gov/media/134922/download). PCR reactions consisted of 45 amplification cycles, and Ct values of any magnitude were deemed positive when represented by a characteristic specific amplification curve. A valid influenza A subtype was contingent on co-detection of the universal influenza type A sequence target. Absence of influenza A and/or B detection in specimens registering RNP Ct values ≥38 was considered inconclusive for the undetected virus(es).

RSV detection by RT-PCR
Total nucleic acid extract from 100 microliters of upper respiratory specimen collected in viral transport medium was prepared using the MagNA Pure LC Total Nucleic Acid Isolation Kit and MagNA Pure 96 automated extraction platform or QIAamp 96 Virus QiaCube HT kit and QiaCube HT automated extraction system. Extracts (100 microliters eluate volume) were tested by RT-PCR using the QuantStudio 3, QuantStudio 5, or QuantStudio 6 Real-Time PCR System for RSV (universal), RSV-A, and RSV-B.

A conserved sequence target in the RSV matrix gene was amplified using methods adapted from published procedures (https://doi.org/10.1016/j.jviromet.2019.113676) and unpublished protocols developed at the CDC. Pan-RSV screening employed AgPath-ID One-Step RT-PCR Reagents (Applied Biosystems), forward primer GGCAAATATGGAAACATACGTGAA (ThermoFisher, Waltham, MA), unpublished CDC reverse primer modified from (https://doi.org/10.1016/j.jviromet.2019.113676) (ThermoFisher), and unpublished CDC fluorescent hydrolysis probe modified from (https://doi.org/10.1016/j.jviromet.2019.113676) (Integrated DNA Technologies, Coralville, IA). RT-PCR reactions contained 12.5 microliters 2x reaction mix, 1.0 micromolar each forward and reverse primer, 0.25 micromolar probe, 1.0 microliter enzyme, and 5 microliters nucleic acid extract in a total volume of 25 microliters nuclease-free H_2O. Cycling conditions consisted of 45°C x 10 min, 95°C x 10 min, and 45 cycles of 95°C x 15 sec followed by 55°C x 60 sec. Ct values of any magnitude were deemed positive when represented by a characteristic specific amplification curve.

Subgroup differentiation of RSV screen-positive specimens employed a CDC method using AgPath-ID One-Step RT-PCR Reagents, unpublished CDC nucleocapsid (N) gene forward primer modified from (https://doi.org/10.1016/j.jviromet.2019.113676) (ThermoFisher), unpublished CDC N gene reverse primer modified from (https://doi.org/10.1016/j.jviromet.2019.113676) (ThermoFisher), and RSV-A N-gene subgroup-specific fluorescent hydrolysis probe <FAM>ACACTCAACAAAGA<BHQ1dT>CAACTTCTRTCATCCAGCA-phosphate (https://doi.org/10.1016/j.jviromet.2019.113676) (Biosearch Technologies, Novato, CA) or

© 2026 Ma KC et al. *JAMA Network Open*.

unpublished CDC RSV-B N-gene fluorescent hydrolysis probe modified from (https://doi.org/10.1016/j.jviromet.2019.113676) (Biosearch). RT-PCR reactions contained 12.5 microliters 2x reaction mix, 1.0 micromolar each forward and reverse primer, 0.25 micromolar probe, 1.0 microliter enzyme, and 5 microliters nucleic acid extract in a total volume of 25 microliters nuclease-free H_2O. Cycling conditions consisted of 45°C x 10 min, 95°C x 10 min, and 45 cycles of 95°C x 15 sec followed by 55°C x 60 sec. Ct values of any magnitude were deemed positive when represented by a characteristic specific amplification curve.

Each specimen also was tested for RNP using TaqPath 1-Step RT-qPCR Master Mix CG (https://www.fda.gov/media/134922/download). A valid RSV subgroup identification was contingent on co-detection of the universal RSV target. Absence of RSV detection in specimens registering RNP Ct values ≥40 was considered inconclusive for RSV RNA. All RT-PCR reactions were performed in single-plex format.

HMPV detection by RT-PCR

Total nucleic acid extract from 100 microliters of upper respiratory specimen collected in viral transport medium was prepared using the MagNA Pure LC Total Nucleic Acid Isolation Kit and MagNA Pure 96 automated extraction platform or QIAamp 96 Virus QiaCube HT kit and QiaCube HT automated extraction system. Extracts (100 microliters eluate volume) were tested by RT-PCR using the QuantStudio 3, QuantStudio 5, or QuantStudio 6 Real-Time PCR System for a conserved sequence target in the hMPV fusion gene (https://doi.org/10.1128/jcm.02270-10)—forward primer CAAGTGTGACATTGCTGAYCTRAA (Biosearch), reverse primer ACTGCC GCACAACATTTAGRAA (Biosearch), and fluorescent hydrolysis probe <FAM>TGGCYGTYAGCTTCAGTCAATTCAACAGA<BHQ-1> (Biosearch)—with AgPath-ID One-Step RT-PCR Reagents. RT-PCR reactions contained 12.5 microliters 2x reaction mix, 1.0 micromolar each forward and reverse primer, 0.25 micromolar probe, 1.0 microliter enzyme, and 5 microliters nucleic acid extract in a total volume of 25 microliters nuclease-free H_2O. Cycling conditions consisted of 45°C x 10 min, 95°C x 10 min, and 45 cycles of 95°C x 15 sec followed by 55°C x 60 sec. Ct values of any magnitude were deemed positive when represented by a characteristic specific amplification curve.

Each specimen also was tested for RNP using TaqPath 1-Step RT-qPCR Master Mix CG (https://www.fda.gov/media/134922/download). Absence of hMPV detection in specimens registering RNP Ct values ≥40 was considered inconclusive for hMPV RNA.

3. Analytic methods

Defining immunocompromising conditions

Immunocompromising conditions included active solid organ or hematologic cancer (defined as newly diagnosed cancer or cancer treatment within the past 6 months); solid organ transplant; bone marrow/stem cell transplant; HIV infection; congenital immunodeficiency syndrome; use of an immunosuppressive medication within the past 30 days; splenectomy; or another condition that causes moderate or severe immunosuppression.

© 2026 Ma KC et al. *JAMA Network Open*.

Bootstrap-based P-values and 95% CIs for differences in VE

We used a non-parametric bootstrapping method to formally assess statistical evidence for differences in VE between in-hospital severe outcomes and hospitalization. Overlapping 95% CIs have been sometimes used as a heuristic to assess differences in VE, but are known to be conservative and do not account for correlation between effect estimates [1]. A bootstrap-based method should better account for the implicit correlation between effect estimates that may arise because in-hospital severe outcomes are nested within hospitalization overall.

Across 10,000 iterations separately for adults ≥18 and ≥65 years, we use the following approach:

1. Draw a bootstrapped dataset with size equal to the original dataset using case-resampling, i.e. sample rows of the original dataset with replacement.

2. Fit separate logistic regression models for the association of 2024–2025 COVID-19 vaccination with COVID-19–associated hospitalization and for each of the severe in-hospital outcomes. Letting β_1 represent the association of vaccination with case-control status on the logit scale and ϕ is a vector of coefficients corresponding to the confounders included in the model, including age, sex, race and ethnicity, geographic region (U.S. Department of Health and Human Services Region), calendar time (biweekly intervals), and Charlson comorbidity index:

   $$logit(case\ status) = \beta_0 + \beta_1 * vaccination + \phi * confounders$$

   VE_1 representing VE against COVID-19–associated hospitalization is therefore given by $100 - 100 * exp\ (\beta_1)$. Similarly, the association of vaccination with case-control status restricting to COVID-19 cases with a given severe in-hospital outcome is given by β_2:

   $$logit(case\ status) = \beta_0 + \beta_2 * vaccination + \phi * confounders$$

   And VE_2 representing VE against the COVID-19–associated severe in-hospital outcome is given by $100 - 100 * exp\ (\beta_2)$. Because the number of cases with 2024–2025 vaccination experiencing IMV receipt or death was low (n = 6), in some bootstrap samples, no vaccinated cases with this outcome were selected causing convergence problems for standard logistic regression. In such cases, we used Firth regression instead to estimate β_2.

3. Calculate the differences $\beta_2^* - \beta_1^*$ and $VE_1^* - VE_2^*$, where * indicates these quantities were derived from bootstrapping. Store the results and proceed to the next iteration.

Using the bootstrapped distribution of $\beta_2^* - \beta_1^*$, we calculated *P*-values by inverting confidence intervals [2]. Under this approach, a series of confidence intervals were calculated at the $1 - \alpha$ level for a range of α values, with the *P*-value defined as the smallest α such that 0 is not contained within the CI for $\beta_2^* - \beta_1^*$. We used bias-corrected and accelerated bootstrap intervals to calculate CIs adjusting for bias and skewness in the bootstrap distribution [3].

© 2026 Ma KC et al. *JAMA Network Open*.

In a sensitivity analysis, we compared results with a different approach used to calculate bootstrap-based *P*-values as described in Fox (2008) [4]. We used the bootstrapped distribution $\beta_2^* - \beta_1^*$ and shifted it by the observed difference in coefficients $\widehat{\beta_1} - \widehat{\beta_2}$ in the original dataset to approximate a null distribution around the null hypothesis of $H_0\colon \beta_2 - \beta_1 = 0$ (see also [5,6]). This method assumed translation invariance of these two distributions. We then calculated the proportion of shifted $\left|(\beta_2^* - \beta_1^*) - (\widehat{\beta_1} - \widehat{\beta_2})\right|$ values that were greater in magnitude than $\left|\widehat{\beta_1} - \widehat{\beta_2}\right|$.

The two approaches relied on different assumptions but appeared to yield generally similar *P*-values (Supplementary Table 1). We also computed and presented 95% CIs for the difference in VE on the percentage scale to help interpret effect sizes.

IVY Network Hospitals

IVY Network hospitals by DHHS region (see the Table in the main article) are as follows: Region 1: Baystate Medical Center (Springfield, Massachusetts), Beth Israel Deaconess Medical Center (Boston, Massachusetts), and Yale University (New Haven, Connecticut); Region 2: Montefiore Medical Center (New York, New York); Region 3: Johns Hopkins Hospital (Baltimore, Maryland); Region 4: Emory University Medical Center (Atlanta, Georgia), University of Miami Medical Center (Miami, Florida), Vanderbilt University Medical Center (Nashville, Tennessee), and Wake Forest University Baptist Medical Center (Winston-Salem, North Carolina); Region 5: Cleveland Clinic (Cleveland, Ohio), Hennepin County Medical Center (Minneapolis, Minnesota), Henry Ford Health (Detroit, Michigan), The Ohio State University Wexner Medical Center (Columbus, Ohio), and University of Michigan Hospital (Ann Arbor, Michigan); Region 6: Baylor Scott & White Medical Center (Temple, Texas) and Baylor University Medical Center (Dallas, Texas); Region 7: Barnes-Jewish Hospital (St. Louis, Missouri) and University of Iowa Hospitals (Iowa City, Iowa); Region 8: Intermountain Medical Center (Murray, Utah), UCHealth University of Colorado Hospital (Aurora, Colorado), and University of Utah (Salt Lake City, Utah); Region 9: Stanford University Medical Center (Stanford, California), Ronald Reagan UCLA Medical Center (Los Angeles, California), and University of Arizona Medical Center (Tucson, Arizona); and Region 10: Oregon Health and Science University Hospital (Portland, Oregon) and University of Washington (Seattle, Washington).

eReferences

1. Knol MJ, Pestman WR, Grobbee DE. The (mis)use of overlap of confidence intervals to assess effect modification. Eur J Epidemiol. 2011;26: 253–254.

2. Thulin M. Modern Statistics with R. [cited 8 Aug 2025]. Available: https://www.modernstatisticswithr.com/

3. Efron B. Better bootstrap confidence intervals. J Am Stat Assoc. 1987;82: 171.

© 2026 Ma KC et al. *JAMA Network Open*.

4. Fox J. Bootstrapping Regression Models. Appendix to An R and S-PLUS Companion to Applied Regression. 2008.

5. Tibshirani R, Hall P, Wilson SR. Bootstrap hypothesis testing. Biometrics. 1992;48: 969–970.

6. Hall P, Wilson SR. Two guidelines for bootstrap hypothesis testing. Biometrics. 1991;47: 757.

© 2026 Ma KC et al. *JAMA Network Open*.

eTable 1. Bootstrap-Based[a] *P* Values and 95% CIs for Differences in the Effectiveness of the 2024-2025 COVID-19 Vaccine Against COVID-19–Associated Hospitalization vs In-Hospital Severe Outcomes – IVY Network, 26 hospitals, September 1, 2024–April 30, 2025.

Age group and outcomes being compared	*P*-value obtained by inverting confidence intervals	*P*-value obtained by shifting bootstrap distribution to generate a null distribution	95% CI for percentage difference in VE against severe outcome minus VE against hospitalization
≥18 years			
Supplemental oxygen therapy versus hospitalization	0.142	0.150	(-2%, 15%)
Acute respiratory failure versus hospitalization	0.413	0.405	(-14%, 28%)
ICU admission versus hospitalization	0.074	0.094	(-2%, 38%)
IMV or death versus hospitalization[b]	0.004	0.043	(18%, 57%)
≥65 years			
Supplemental oxygen therapy versus hospitalization	0.458	0.465	(-5%, 12%)
Acute respiratory failure versus hospitalization	0.679	0.670	(-35%, 16%)
ICU admission versus hospitalization	0.420	0.391	(-17%, 31%)
IMV or death versus hospitalization	0.093	0.116	(-3%, 47%)

Abbreviations: CI, confidence interval; COVID-19, coronavirus disease 2019; IVY, Investigating Respiratory Viruses in the Acutely Ill.

[a] To test the hypothesis that VE differed by outcome, *P*-values and 95% CIs for differences in VE against hospitalization versus VE against severe outcomes were calculated using bootstrapping with 10,000 replicates. In each iteration, the difference in regression coefficients between a severe outcome and hospitalization was computed. We used Firth regression in rare iterations when no vaccinated cases experiencing IMV receipt or death (n = 6) were sampled, which caused convergence problems for standard logistic regression. We calculated *P*-values using

© 2026 Ma KC et al. *JAMA Network Open*.

the bootstrap replicates by inverting confidence intervals [2] and by a method described by Fox for approximating the null distribution (2008) [4].

[b] indicates *P*-value for difference in regression coefficients is < 0.05 using both methods.

© 2026 Ma KC et al. *JAMA Network Open*.

eTable 2. Number of COVID-19 Cases by Hospital Admission Week and SARS-COV-2 Lineage – IVY Network, 26 hospitals, September 1, 2024–April 30, 2025.

Week	24A (JN.1)	24B (JN.1.11.1)	24C (KP.3)	24E (KP.3.1.1)	24F (XEC)	24G (KP.2.3)	24D (XDV.1)	24H (LF.7)	25A (LP.8.1)	25C (XFG)
9/1/2024	7	8	5	18	4	14	0	0	0	0
9/8/2024	7	4	8	36	5	4	0	0	0	0
9/15/2024	9	5	9	27	2	4	0	0	0	0
9/22/2024	8	5	5	25	1	6	0	0	0	0
9/29/2024	5	3	2	24	3	2	1	0	0	0
10/6/2024	3	7	1	18	3	4	0	0	0	0
10/13/2024	0	0	3	16	10	3	1	0	0	0
10/20/2024	1	0	0	17	4	2	0	0	0	0
10/27/2024	1	0	2	16	12	1	0	3	1	0
11/3/2024	1	2	1	7	9	0	0	1	0	0
11/10/2024	2	0	2	14	6	1	0	1	2	0
11/17/2024	1	0	1	15	11	0	0	3	0	0
11/24/2024	3	1	0	16	8	0	0	0	1	0
12/1/2024	1	1	0	10	13	1	0	3	5	0
12/8/2024	1	1	4	8	14	1	0	0	0	0
12/15/2024	1	0	0	5	16	0	0	0	1	0
12/22/2024	3	0	0	8	6	0	0	1	5	0
12/29/2024	0	0	0	9	17	0	0	1	2	0
1/5/2025	2	0	0	6	11	0	0	1	4	0
1/12/2025	1	0	0	8	9	0	0	0	2	0
1/19/2025	0	0	0	5	14	0	0	0	2	0
1/26/2025	1	0	0	2	5	0	0	0	4	0
2/2/2025	0	0	0	6	7	0	0	0	2	0
2/9/2025	0	0	0	5	5	0	0	1	8	0
2/16/2025	0	0	0	4	1	0	0	2	4	0
2/23/2025	0	0	0	4	2	0	0	0	12	0
3/2/2025	1	0	0	5	1	0	1	2	7	0
3/9/2025	0	0	0	1	2	0	0	1	8	0
3/16/2025	1	0	0	1	4	0	0	0	5	0
3/23/2025	2	0	0	4	2	0	0	2	11	0
3/30/2025	1	0	0	2	7	0	0	2	12	1
4/6/2025	0	0	0	1	0	0	0	1	8	1
4/13/2025	1	0	1	1	1	0	0	3	10	0
4/20/2025	0	0	0	4	3	0	0	0	14	0
4/27/2025	0	0	0	0	0	0	0	0	4	1

© 2026 Ma KC et al. *JAMA Network Open*.

Abbreviations: COVID-19, coronavirus disease 2019; IVY, Investigating Respiratory Viruses in the Acutely Ill; SARS-CoV-2, severe acute respiratory syndrome coronavirus 2.

Dates are for the start of the admission week. SARS-CoV-2 lineage was identified using Nextstrain after conducting viral whole-genome sequencing.

© 2026 Ma KC et al. *JAMA Network Open*.

eTable 3. Counts of Spike Protein N-Terminal Domain Substitutions or Deletion by Nextstrain Clade – IVY Network, 26 hospitals, September 1, 2024–April 30, 2025.

N-terminal domain substitution or deletion	Nextstrain clade
**None (n = 62)**	• 24C (KP.3) [n = 37]<br>• 24B (JN.1.11.1) [n = 11]<br>• 24A (JN.1) [n = 8]<br>• 25C (XFG) [n = 3]<br>• 24D (XDV.1) [n = 2]<br>• 24E (KP.3.1.1) [n = 1]
**T22N and F59S substitutions (n = 237)**	• 24F (XEC) [n = 217]<br>• recombinant [n = 17]<br>• 24A (JN.1) [n = 1]<br>• 24B (JN.1.11.1) [n = 1]<br>• 24D (XDV.1) [n = 1]
**T22N substitution[a] (n = 290)**	• 24F (XEC) [n = 217]<br>• 24H (LF.7) [n = 28]<br>• recombinant [n = 21]<br>• 24A (JN.1) [n = 12]<br>• 25A (LP.8.1) [n = 4]<br>• 24C (KP.3) [n = 3]<br>• 24B (JN.1.11.1) [n = 2]<br>• 24D (XDV.1) [n = 1]<br>• 24E (KP.3.1.1) [n = 1]<br>• 24G (KP.2.3) [n = 1]
**S31 deletion[a] (n = 602)**	• 24E (KP.3.1.1) [n = 347]<br>• 25A (LP.8.1) [n = 134]<br>• 24G (KP.2.3) [n = 43]<br>• 24A (JN.1) [n = 41]<br>• 24B (JN.1.11.1) [n = 21]<br>• recombinant [n = 8]<br>• 24C (KP.3) [n = 7]<br>• 24F (XEC) [n = 1]

Abbreviations: IVY, Investigating Respiratory Viruses in the Acutely Ill; SARS-CoV-2, severe acute respiratory syndrome coronavirus 2.

[a] includes n = 9 SARS-CoV-2 strains with both the S31 deletion and T22N substitution.

© 2026 Ma KC et al. *JAMA Network Open*.

eFigure 1. Participant Flow Diagram and Analytic Cohorts

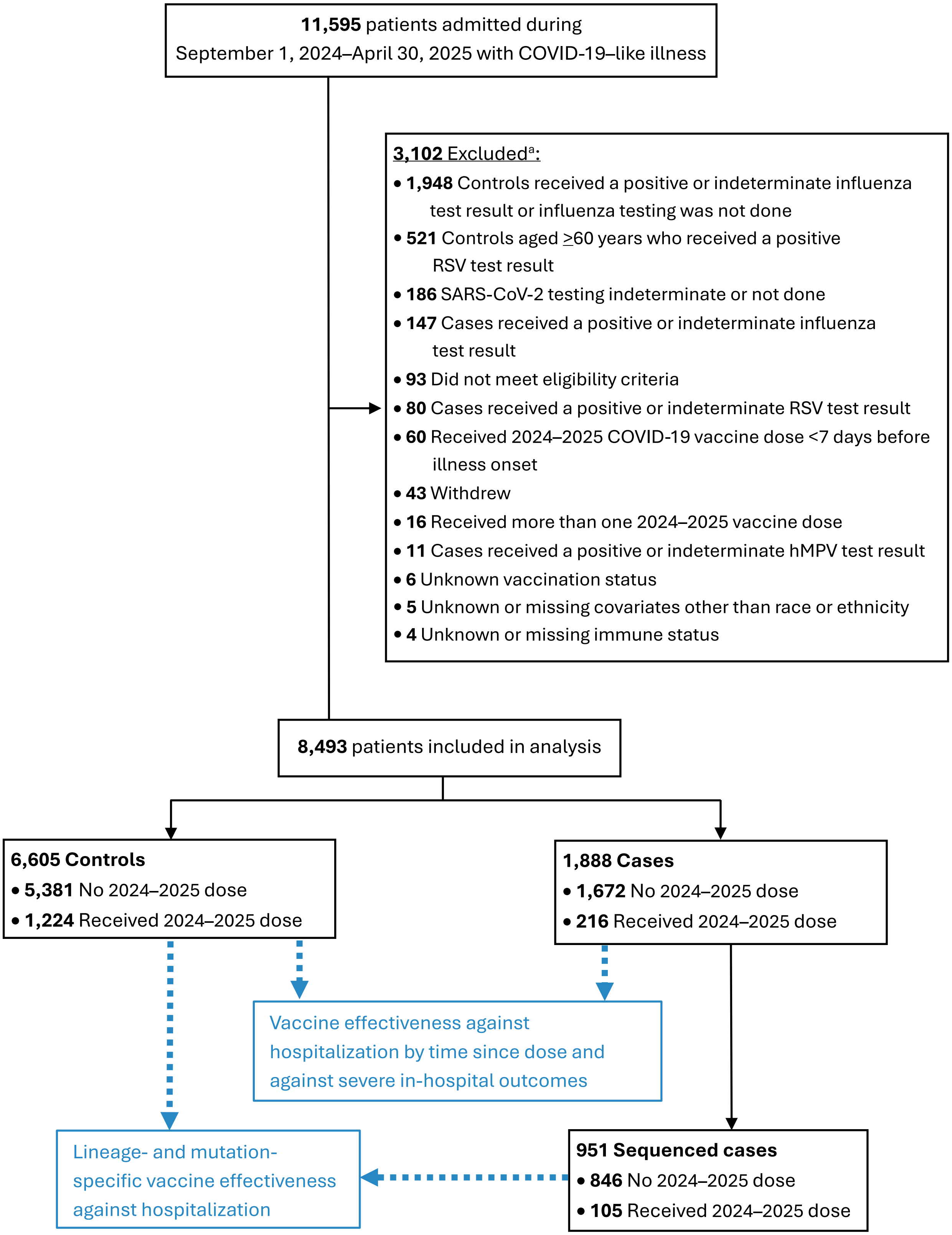

[a] Exclusions are not mutually exclusive.

© 2026 Ma KC et al. *JAMA Network Open*.

eFigure 2. Numbers of COVID-19 Case and Test-Negative Control Participants by Admission Week – IVY Network, 26 hospitals, September 1, 2024–April 30, 2025.

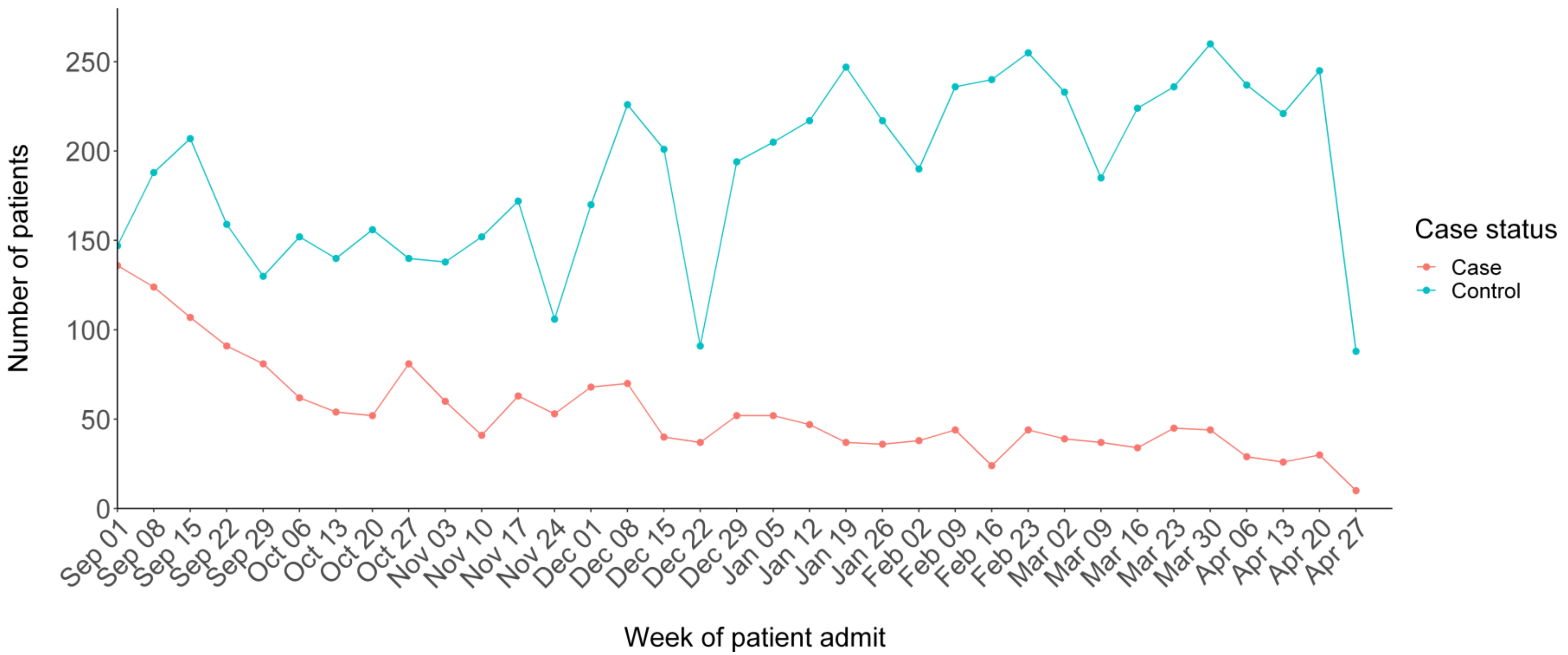

Abbreviations: COVID-19, coronavirus disease 2019; IVY, Investigating Respiratory Viruses in the Acutely Ill.

Dates are for the start of the admission week.

© 2026 Ma KC et al. *JAMA Network Open*.

eFigure 3. 2024-2025 COVID-19 Vaccination Coverage by Admission Week Among Test-Negative Control Participants – IVY Network, 26 hospitals, September 1, 2024–April 30, 2025.[a]

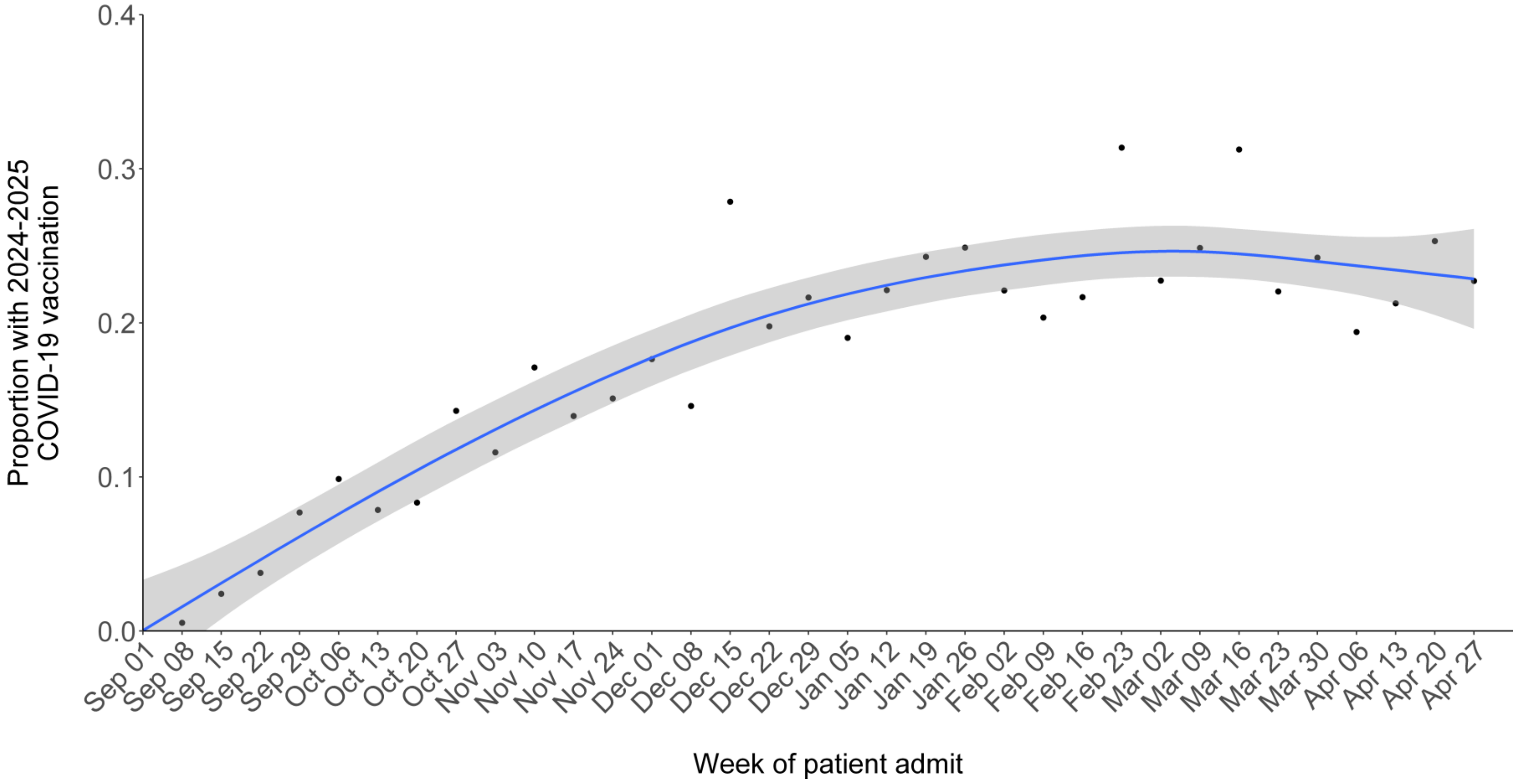

Abbreviations: COVID-19, coronavirus disease 2019; IVY, Investigating Respiratory Viruses in the Acutely Ill.

[a] Locally estimated scatterplot smoothing (LOESS) was used to smooth the weekly data and generate 95% confidence intervals.

© 2026 Ma KC et al. *JAMA Network Open*.

Dates are for the start of the admission week.

© 2026 Ma KC et al. *JAMA Network Open*.

eFigure 4. Effectiveness of 2024-2025 COVID-19 Vaccine Against COVID-19–Associated Hospitalization Among Immunocompetent Adults by Time Since Dose Receipt (60-Day Windows) and Age Group – IVY Network, 26 hospitals, September 1, 2024–April 30, 2025.

Age group and days since receipt of 2024–2025 COVID-19 vaccine	No. of vaccinated cases/Total cases, n (%)	No. of vaccinated controls/Total controls, n (%)	Median interval since dose among vaccinated cases[a], days (IQR)	Median interval since dose among vaccinated controls[a], days (IQR)	Vaccine effectiveness[b], % (95% CI)
**≥18 years**					
Received 2024-2025 COVID-19 dose **≥7 days earlier**	153/1,435 (11)	850/4,696 (18)	80 (43–137)	108 (66–151)	**40 (27–51)**
2024-2025 COVID-19 dose, **7–59 days earlier**	56/1,338 (4)	183/4,029 (5)	33 (20–48)	31 (19–46)	**30 (5–50)**
2024-2025 COVID-19 dose, **60–119 days earlier**	43/1,325 (3)	300/4,146 (7)	82 (70–97)	91 (74–106)	**51 (32–66)**
2024-2025 COVID-19 dose, **120–179 days earlier**	36/1,318 (3)	273/4,119 (7)	143 (130–160)	147 (131–161)	**43 (18–61)**
2024-2025 COVID-19 dose, **≥180 days earlier**	18/1,300 (1)	94/3,940 (2)	197 (191–210)	195 (186–207)	**11 (-49 to 50)[c]**
**≥65 years**					
Received 2024-2025 COVID-19 dose **≥7 days earlier**	127/962 (13)	608/2,488 (24)	82 (49–137)	109 (67–155)	**45 (31–56)**
2024-2025 COVID-19 dose, **7–59 days earlier**	43/878 (5)	126/2,006 (6)	34 (23–49)	32 (20–45)	**43 (18–61)**
2024-2025 COVID-19 dose, **60–119 days earlier**	37/872 (4)	212/2,092 (10)	80 (69–100)	91 (74–105)	**54 (33–69)**
2024-2025 COVID-19 dose, **120–179 days earlier**	32/867 (4)	193/2,073 (9)	141 (128–158)	149 (133–163)	**41 (12–62)**
2024-2025 COVID-19 dose, **≥180 days earlier**	15/850 (2)	77/1,957 (4)	197 (194–214)	197 (188–208)	**26 (-31 to 61)[c]**

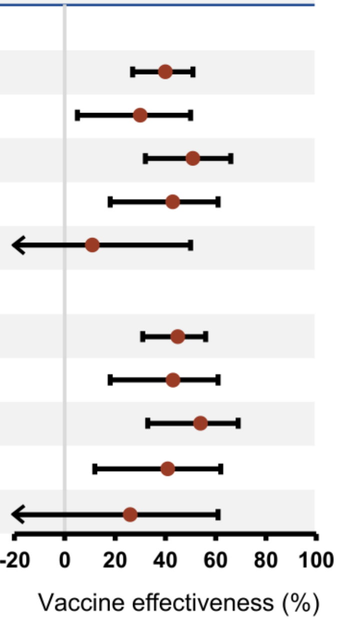

Abbreviations: CI, confidence interval; COVID-19, coronavirus disease 2019; IQR, interquartile range; IVY, Investigating Respiratory Viruses in the Acutely Ill; SARS-CoV-2, severe acute respiratory syndrome coronavirus 2.

[a] Time since vaccination with a 2024–2025 COVID-19 vaccine.

© 2026 Ma KC et al. *JAMA Network Open*.

[b] Vaccine effectiveness was calculated by comparing the odds of 2024–2025 COVID-19 vaccination in cases and controls using the equation: (1 – adjusted odds ratio) x 100%. Odds ratios were estimated by multivariable logistic regression adjusted for age, sex, race and ethnicity, geographic region (U.S. Department of Health and Human Services Region), calendar time (biweekly intervals), and Charlson comorbidity index.

[c] Estimates are imprecise due to limited numbers of enrolled patients with dose receipt ≥180 days earlier than hospitalization.

© 2026 Ma KC et al. *JAMA Network Open*.

eFigure 5. Effectiveness of 2024-2025 COVID-19 Vaccine Against COVID-19–Associated Hospitalization Among Immunocompromised[a] Adults by Age Group – IVY Network, 26 hospitals, September 1, 2024–April 30, 2025.

Age group and days since receipt of 2024–2025 COVID-19 vaccine	No. of vaccinated cases/Total cases, n (%)	No. of vaccinated controls/Total controls, n (%)	Median interval since dose among vaccinated cases[b], days (IQR)	Median interval since dose among vaccinated controls[b], days (IQR)	Vaccine effectiveness[c], % (95% CI)
**≥18 years**					
Received 2024-2025 COVID-19 dose **≥7 days earlier**	63/453 (14)	374/1,909 (20)	79 (49–129)	105 (60–148)	**23 (-5 to 44)**
**≥65 years**					
Received 2024-2025 COVID-19 dose **≥7 days earlier**	47/260 (18)	248/939 (26)	79 (49–131)	106 (56–153)	**36 (6–57)**

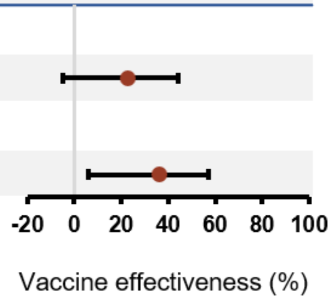

Abbreviations: CI, confidence interval; COVID-19, coronavirus disease 2019; IQR, interquartile range; IVY, Investigating Respiratory Viruses in the Acutely Ill; SARS-CoV-2, severe acute respiratory syndrome coronavirus 2.

[a] Immunocompromising conditions included active solid tumor or hematologic malignancy (defined as newly diagnosed malignancy or treatment within the past 6 months); solid organ transplant; hematopoietic cell transplant; HIV infection; congenital immunodeficiency syndrome; use of an immunosuppressive medication within the past 30 days; splenectomy; or another condition that causes moderate or severe immunosuppression.

[b] Time since vaccination with a 2024–2025 COVID-19 vaccine.

[c] Vaccine effectiveness was calculated by comparing the odds of 2024–2025 COVID-19 vaccination in cases and controls using the equation: (1 – adjusted odds ratio) x 100%. Odds ratios were estimated by multivariable logistic regression adjusted for age, sex, race and ethnicity, geographic region (U.S. Department of Health and Human Services Region), calendar time (biweekly intervals), and Charlson comorbidity index.

© 2026 Ma KC et al. *JAMA Network Open*.

eFigure 6. Effectiveness of 2024-2025 COVID-19 Vaccine Against COVID-19–Associated Hospitalization Among Case Participants With SARS-CoV-2 Lineage Successfully Identified Through Whole-Genome Sequencing and Among All Case Participants by Time Since Dose Receipt – IVY Network, 26 hospitals, September 1, 2024–April 30, 2025.

Cohort and days since receipt of 2024–2025 COVID-19 vaccine	No. of vaccinated cases/Total cases, n (%)	No. of vaccinated controls/Total controls, n (%)	Median interval since dose among vaccinated cases[a], days (IQR)	Median interval since dose among vaccinated controls[a], days (IQR)	Vaccine effectiveness[b], % (95% CI)
**≥18 years**					
Received 2024-2025 COVID-19 dose **≥7 days earlier**	216/1,888 (11)	1,224/6,605 (19)	80 (45–136)	108 (64–150)	**36 (24–46)**
2024-2025 COVID-19 dose, **7–89 days earlier**	118/1,790 (7)	494/5,875 (8)	50 (27–69)	53 (31–72)	**33 (17–47)**
2024-2025 COVID-19 dose, **90–179 days earlier**	76/1,748 (4)	593/5,974 (10)	130 (113–154)	131 (111–153)	**42 (26–56)**
2024-2025 COVID-19 dose, **≥180 days earlier**	22/1,694 (1)	137/5,518 (2)	197 (191–210)	195 (188–208)	**14 (-36 to 48)[d]**
**≥18 years, sequenced COVID-19 case-patients[c]**					
Received 2024-2025 COVID-19 dose **≥7 days earlier**	105/951 (11)	1,224/6,605 (19)	88 (51–135)	108 (64–150)	**41 (26–53)**
2024-2025 COVID-19 dose, **7–89 days earlier**	54/900 (6)	494/5,875 (8)	52 (33–68)	53 (31–72)	**45 (26–60)**
2024-2025 COVID-19 dose, **90–179 days earlier**	41/887 (5)	593/5,974 (10)	129 (116–151)	131 (111–153)	**36 (11–56)**
2024-2025 COVID-19 dose, **≥180 days earlier**	10/856 (1)	137/5,518 (2)	197 (191–211)	195 (188–208)	**34 (-24 to 69)[d]**

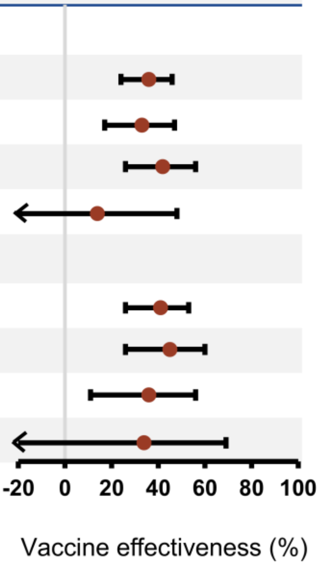

Abbreviations: CI, confidence interval; COVID-19, coronavirus disease 2019; IQR, interquartile range; IVY, Investigating Respiratory Viruses in the Acutely Ill; SARS-CoV-2, severe acute respiratory syndrome coronavirus 2.

[a] Time since vaccination with a 2024–2025 COVID-19 vaccine.

[b] Vaccine effectiveness was calculated by comparing the odds of 2024–2025 COVID-19 vaccination in cases and controls using the equation: (1 – adjusted odds ratio) x 100%. Odds ratios were estimated by multivariable logistic regression adjusted for age, sex,

© 2026 Ma KC et al. *JAMA Network Open*.

race and ethnicity, geographic region (U.S. Department of Health and Human Services Region), calendar time (biweekly intervals), and Charlson comorbidity index.

[c] SARS-CoV-2 whole-genome sequences were considered adequate if they had a Nextclade completeness score greater than 80 and Nextclade quality control status of "good" or "mediocre."

[d] Estimates are imprecise due to limited numbers of enrolled patients with dose receipt ≥180 days earlier than hospitalization.

© 2026 Ma KC et al. *JAMA Network Open*.

eFigure 7. Number of COVID-19 Case by Hospital Admission Week and SARS-CoV-2 Spike Protein N-Terminal Domain Substitutions and Deletions – IVY Network, 26 hospitals, September 1, 2024–April 30, 2025.[a]

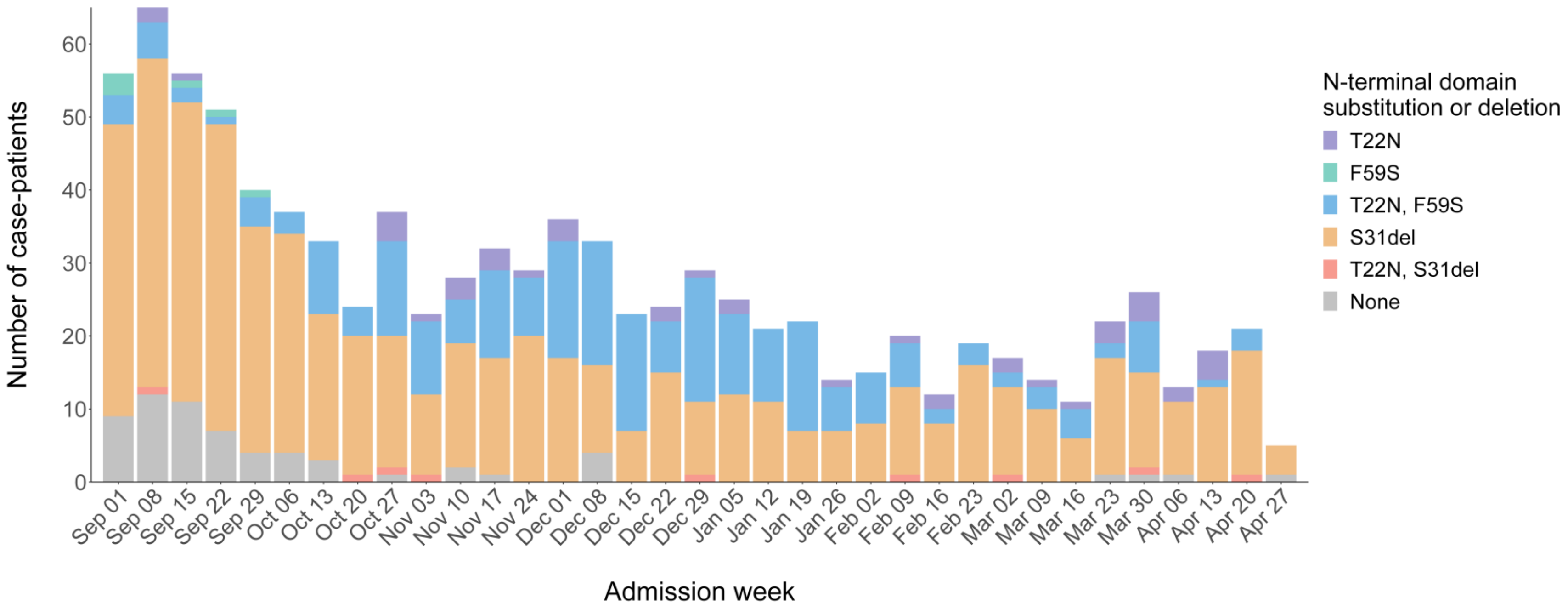

Abbreviations: COVID-19, coronavirus disease 2019; IVY, Investigating Respiratory Viruses in the Acutely Ill; SARS-CoV-2, severe acute respiratory syndrome coronavirus 2.

[a] Dates are for the start of the admission week. SARS-CoV-2 spike protein substitutions and deletions were identified after conducting viral whole-genome sequencing.

© 2026 Ma KC et al. *JAMA Network Open*.
