# Supplementary material for: Estimated Effectiveness of 2024-2025 COVID-19 Vaccination Against Severe COVID-19
Source: JAMA Netw Open. 2026 Feb 3;9(2):e2557415. doi: 10.1001/jamanetworkopen.2025.57415 (PMC12869339; doi:10.1001/jamanetworkopen.2025.57415)
Supplement: Supplement 2. — Nonauthor Collaborators [file jamanetwopen-e2557415-s002.pdf]

Supplemental Online Content: Nonauthor Collaborators
*First name, last name, and suffix (if applicable) are required and will appear in PubMed.

***Group Name(s): Investigating Respiratory Viruses in the Acutely Ill (IVY) Network**							
***First Name and Middle Initial(s)**	***Last Name**	***Suffix (eg, Jr, III)**	Academic Degrees	Institution	Location (city, state/province, country)	Role or Contribution, eg, chair, principal investigator	Group (if more than 1 Group listed in the byline) and/or Subgroup (eg, Steering Committee)
Tresa	McNeal			Baylor, Scott and White	Temple, Texas	Site Contributions	IVY Network member
Nicole	Calhoun			Baylor, Scott and White	Temple, Texas	Site Contributions	IVY Network member
Jocelyn	Cravens			Baylor, Scott and White	Temple, Texas	Site Contributions	IVY Network member
Kempapura	Murthy			Baylor, Scott and White	Temple, Texas	Site Contributions	IVY Network member
Leah	Odame-Bamfo			Baylor, Scott and White	Temple, Texas	Site Contributions	IVY Network member
Spencer	Rose			Baylor, Scott and White	Temple, Texas	Site Contributions	IVY Network member
Michael	Smith			Baylor, Scott and White	Temple, Texas	Site Contributions	IVY Network member
Barbara	Hairston			Baylor, Scott and White	Temple, Texas	Site Contributions	IVY Network member
Amanda	McKillop			Baylor, Scott and White	Temple, Texas	Site Contributions	IVY Network member
Victoria Harkins	Walston			Baylor, Scott and White	Temple, Texas	Site Contributions	IVY Network member
Robert L.	Gottlieb			Baylor, Scott and White	Dallas, Texas	Site Contributions	IVY Network member
Catherine	Raver			Baylor, Scott and White	Dallas, Texas	Site Contributions	IVY Network member
Sydney	Buehrig			Baylor, Scott and White	Dallas, Texas	Site Contributions	IVY Network member
Priyanka	Rana			Baylor, Scott and White	Dallas, Texas	Site Contributions	IVY Network member
Ashley	Bychkowski			Baylor, Scott and White	Dallas, Texas	Site Contributions	IVY Network member
Symone	Dunkley			Baylor, Scott and White	Dallas, Texas	Site Contributions	IVY Network member
Denisse	Mariscal			Baylor, Scott and White	Dallas, Texas	Site Contributions	IVY Network member
Tammy	Fisher			Baylor, Scott and White	Dallas, Texas	Site Contributions	IVY Network member
Daniela	Gonzalez			Baylor, Scott and White	Dallas, Texas	Site Contributions	IVY Network member
Therissa	Grefsrud			Baylor, Scott and White	Dallas, Texas	Site Contributions	IVY Network member
Mariana	Hurutado-Rodriguez			Baylor, Scott and White	Dallas, Texas	Site Contributions	IVY Network member
Gabriela	Perez			Baylor, Scott and White	Dallas, Texas	Site Contributions	IVY Network member
Jay	Steingrub			Baystate Medical Center	Springfield, Massachusetts	Site Contributions	IVY Network member
Lesley	De Souza			Baystate Medical Center	Springfield, Massachusetts	Site Contributions	IVY Network member
Scott	Ouellette			Baystate Medical Center	Springfield, Massachusetts	Site Contributions	IVY Network member
Cynthia	Kardos			Baystate Medical Center	Springfield, Massachusetts	Site Contributions	IVY Network member
Rae Lynn	Defeo			Baystate Medical Center	Springfield, Massachusetts	Site Contributions	IVY Network member
Nathan I.	Shapiro			Beth Israel Medical Center	Boston, Massachusetts	Site Contributions	IVY Network member
Michael	Bolstad			Beth Israel Medical Center	Boston, Massachusetts	Site Contributions	IVY Network member
Brianna	Coviello			Beth Israel Medical Center	Boston, Massachusetts	Site Contributions	IVY Network member
Robert	Ciottone			Beth Israel Medical Center	Boston, Massachusetts	Site Contributions	IVY Network member
Arnaldo	Devilla			Beth Israel Medical Center	Boston, Massachusetts	Site Contributions	IVY Network member
Ana	Grafals			Beth Israel Medical Center	Boston, Massachusetts	Site Contributions	IVY Network member
Conor	Higgins			Beth Israel Medical Center	Boston, Massachusetts	Site Contributions	IVY Network member

Supplemental Online Content: Nonauthor Collaborators
*First name, last name, and suffix (if applicable) are required and will appear in PubMed.

***First Name and Middle Initial(s)**	***Last Name**	***Suffix (eg, Jr, III)**	Academic Degrees	Institution	Location (city, state/province, country)	Role or Contribution, eg, chair, principal investigator	Group (if more than 1 Group listed in the byline) and/or Subgroup (eg, Steering Committee)
Carlo	Ottanelli			Beth Israel Medical Center	Boston, Massachusetts	Site Contributions	IVY Network member
Kimberly	Redman			Beth Israel Medical Center	Boston, Massachusetts	Site Contributions	IVY Network member
Douglas	Scaffidi			Beth Israel Medical Center	Boston, Massachusetts	Site Contributions	IVY Network member
Alexander	Weingart			Beth Israel Medical Center	Boston, Massachusetts	Site Contributions	IVY Network member
Omar	Mehkri			Cleveland Clinic	Cleveland, Ohio	Site Contributions	IVY Network member
Megan	Mitchell			Cleveland Clinic	Cleveland, Ohio	Site Contributions	IVY Network member
Zachary	Griffith			Cleveland Clinic	Cleveland, Ohio	Site Contributions	IVY Network member
Connery	Brennan			Cleveland Clinic	Cleveland, Ohio	Site Contributions	IVY Network member
Kiran	Ashok			Cleveland Clinic	Cleveland, Ohio	Site Contributions	IVY Network member
Bryan	Poynter			Cleveland Clinic	Cleveland, Ohio	Site Contributions	IVY Network member
Laurence	Busse			Emory University	Atlanta, Georgia	Site Contributions	IVY Network member
William	Bender			Emory University	Atlanta, Georgia	Site Contributions	IVY Network member
Caitlin	ten Lohuis			Emory University	Atlanta, Georgia	Site Contributions	IVY Network member
Laurynn	Giles			Hennepin County Medical Center	Minneapolis, Minnesota	Site Contributions	IVY Network member
Mary	O'Rourke			Hennepin County Medical Center	Minneapolis, Minnesota	Site Contributions	IVY Network member
Anne	Frosch			Hennepin County Medical Center	Minneapolis, Minnesota	Site Contributions	IVY Network member
Audrey	Hendrickson			Hennepin County Medical Center	Minneapolis, Minnesota	Site Contributions	IVY Network member
Julianna	Prohofsky			Hennepin County Medical Center	Minneapolis, Minnesota	Site Contributions	IVY Network member
Kowsar	Hurreh			Hennepin County Medical Center	Minneapolis, Minnesota	Site Contributions	IVY Network member
Joanna	Kuo			Hennepin County Medical Center	Minneapolis, Minnesota	Site Contributions	IVY Network member
Gina	Maki			Henry Ford Health	Detroit, Michigan	Site Contributions	IVY Network member
Jaskiran	Bansal			Henry Ford Health	Detroit, Michigan	Site Contributions	IVY Network member
Michael	Garcia			Henry Ford Health	Detroit, Michigan	Site Contributions	IVY Network member
Alycia	Lilla			Henry Ford Health	Detroit, Michigan	Site Contributions	IVY Network member
Catherine	McKeon			Henry Ford Health	Detroit, Michigan	Site Contributions	IVY Network member
Maria	Santana-Garces			Henry Ford Health	Detroit, Michigan	Site Contributions	IVY Network member
Tanisha	Shack			Henry Ford Health	Detroit, Michigan	Site Contributions	IVY Network member
Sindhuja	Koneru			Henry Ford Health	Detroit, Michigan	Site Contributions	IVY Network member
Shruti	Tirumala			Henry Ford Health	Detroit, Michigan	Site Contributions	IVY Network member
Joslyn	Bassett			Intermountain Medical Center	Murray, Utah	Site Contributions	IVY Network member
Shandi	Poulson			Intermountain Medical Center	Murray, Utah	Site Contributions	IVY Network member
Vineela	Thumma			Intermountain Medical Center	Murray, Utah	Site Contributions	IVY Network member
Minh	Phan			Johns Hopkins University	Baltimore, Maryland	Site Contributions	IVY Network member
Safa	Saeed			Johns Hopkins University	Baltimore, Maryland	Site Contributions	IVY Network member
Sarah	Karow			Ohio State Medical Center	Columbus, Ohio	Site Contributions	IVY Network member

Supplemental Online Content: Nonauthor Collaborators
*First name, last name, and suffix (if applicable) are required and will appear in PubMed.

*First Name and Middle Initial(s)	*Last Name	*Suffix (eg, Jr, III)	Academic Degrees	Institution	Location (city, state/province, country)	Role or Contribution, eg, chair, principal investigator	Group (if more than 1 Group listed in the byline) and/or Subgroup (eg, Steering Committee)
Maryiam	Khan			Ohio State Medical Center	Columbus, Ohio	Site Contributions	IVY Network member
Gabrielle	Swoope			Ohio State Medical Center	Columbus, Ohio	Site Contributions	IVY Network member
Brooke	Lee			Ohio State Medical Center	Columbus, Ohio	Site Contributions	IVY Network member
Amane	Rasul			Ohio State Medical Center	Columbus, Ohio	Site Contributions	IVY Network member
Kirstina	Luikart			Ohio State Medical Center	Columbus, Ohio	Site Contributions	IVY Network member
Tyler	Haley			Ohio State Medical Center	Columbus, Ohio	Site Contributions	IVY Network member
Amer	Charif			Ohio State Medical Center	Columbus, Ohio	Site Contributions	IVY Network member
Thomas	Sturges			Ohio State Medical Center	Columbus, Ohio	Site Contributions	IVY Network member
Ibrahim	Abu Hammad			Ohio State Medical Center	Columbus, Ohio	Site Contributions	IVY Network member
Rashil	Madan			Ohio State Medical Center	Columbus, Ohio	Site Contributions	IVY Network member
Jenny	Chan			Oregon Health and Sciences University	Portland, Oregon	Site Contributions	IVY Network member
Connie	Tran			Oregon Health and Sciences University	Portland, Oregon	Site Contributions	IVY Network member
Davika	Dige			Oregon Health and Sciences University	Portland, Oregon	Site Contributions	IVY Network member
Anabelle	Blue			Oregon Health and Sciences University	Portland, Oregon	Site Contributions	IVY Network member
Amy	Segura			Oregon Health and Sciences University	Portland, Oregon	Site Contributions	IVY Network member
Riya	Matthew			Oregon Health and Sciences University	Portland, Oregon	Site Contributions	IVY Network member
Adrian	Hernandez			Oregon Health and Sciences University	Portland, Oregon	Site Contributions	IVY Network member
Emily	Tribbett			Oregon Health and Sciences University	Portland, Oregon	Site Contributions	IVY Network member
Genesis	Briceno			Oregon Health and Sciences University	Portland, Oregon	Site Contributions	IVY Network member
Grace Kyin-Ye	Tam			Stanford University	Stanford, California	Site Contributions	IVY Network member
Vanessa	Pitre			Stanford University	Stanford, California	Site Contributions	IVY Network member
Samantha	Ferguson			Stanford University	Stanford, California	Site Contributions	IVY Network member
Cynthia	Perez			Stanford University	Stanford, California	Site Contributions	IVY Network member
Alexandra June	Gordon			Stanford University	Stanford, California	Site Contributions	IVY Network member
Leonard	Basobas			Stanford University	Stanford, California	Site Contributions	IVY Network member
Lily	Lau			Stanford University	Stanford, California	Site Contributions	IVY Network member
Cameron	Hypes			University of Arizona	Tucson, Arizona	Site Contributions	IVY Network member
Karen	Lutrick			University of Arizona	Tucson, Arizona	Site Contributions	IVY Network member
Beth Salvagio	Campbell			University of Arizona	Tucson, Arizona	Site Contributions	IVY Network member
Cody	Tran			University of California-Los Angeles	Los Angeles, California	Site Contributions	IVY Network member
Sukantha	Chandrasekaran			University of California-Los Angeles	Los Angeles, California	Site Contributions	IVY Network member
Omai	Garner			University of California-Los Angeles	Los Angeles, California	Site Contributions	IVY Network member
Samantha	Simon			University of Colorado	Aurora, Colorado	Site Contributions	IVY Network member
Amanda	Martinez			University of Colorado	Aurora, Colorado	Site Contributions	IVY Network member
Amy	Sullivan			University of Colorado	Aurora, Colorado	Site Contributions	IVY Network member

Supplemental Online Content: Nonauthor Collaborators
*First name, last name, and suffix (if applicable) are required and will appear in PubMed.

*First Name and Middle Initial(s)	*Last Name	*Suffix (eg, Jr, III)	Academic Degrees	Institution	Location (city, state/province, country)	Role or Contribution, eg, chair, principal investigator	Group (if more than 1 Group listed in the byline) and/or Subgroup (eg, Steering Committee)
Laura	Aguilar-Marquez			University of Colorado	Aurora, Colorado	Site Contributions	IVY Network member
Erika	Alor			University of Colorado	Aurora, Colorado	Site Contributions	IVY Network member
Yvette	Evans			University of Colorado	Aurora, Colorado	Site Contributions	IVY Network member
Jacob	Rademacher			University of Colorado	Aurora, Colorado	Site Contributions	IVY Network member
Nicholas	Mohr			University of Iowa	Iowa City, Iowa	Site Contributions	IVY Network member
Anne	Zepeski			University of Iowa	Iowa City, Iowa	Site Contributions	IVY Network member
Paul	Nassar			University of Iowa	Iowa City, Iowa	Site Contributions	IVY Network member
Noble	Briggs			University of Iowa	Iowa City, Iowa	Site Contributions	IVY Network member
Jacob	Hampton			University of Iowa	Iowa City, Iowa	Site Contributions	IVY Network member
Cathy	Fairfield			University of Iowa	Iowa City, Iowa	Site Contributions	IVY Network member
Carolina	Rivas			University of Miami	Miami, Florida	Site Contributions	IVY Network member
Weronika Damek	Valvano			University of Michigan	Ann Arbor, Michigan	Site Contributions	IVY Network member
Anne	Kaniclides			University of Michigan	Ann Arbor, Michigan	Site Contributions	IVY Network member
Rebecca	Fong			University of Michigan	Ann Arbor, Michigan	Site Contributions	IVY Network member
Mildred	Wallace			University of Michigan	Ann Arbor, Michigan	Site Contributions	IVY Network member
Chiraag	Balsara			University of Michigan	Ann Arbor, Michigan	Site Contributions	IVY Network member
Rachel	Truson			University of Michigan	Ann Arbor, Michigan	Site Contributions	IVY Network member
Regina	Lehmann			University of Michigan	Ann Arbor, Michigan	Site Contributions	IVY Network member
Abigail	Carolan			University of Michigan	Ann Arbor, Michigan	Site Contributions	IVY Network member
Izza	Imran			University of Michigan	Ann Arbor, Michigan	Site Contributions	IVY Network member
Jozyan	Ujmaya			University of Michigan	Ann Arbor, Michigan	Site Contributions	IVY Network member
William J.	Fitzsimmons			University of Michigan	Ann Arbor, Michigan	Site Contributions	IVY Network member
Rylie	McBride			University of Utah	Salt Lake City, Utah	Site Contributions	IVY Network member
Bryce	Bosworth			University of Utah	Salt Lake City, Utah	Site Contributions	IVY Network member
Bryce	Heslop			University of Utah	Salt Lake City, Utah	Site Contributions	IVY Network member
Joshua	Acidera			University of Washington	Seattle, Washington	Site Contributions	IVY Network member
Maile	Mckeown			University of Washington	Seattle, Washington	Site Contributions	IVY Network member
Leenay	Coughlin			University of Washington	Seattle, Washington	Site Contributions	IVY Network member
Frances	Nagore			University of Washington	Seattle, Washington	Site Contributions	IVY Network member
Dylan	Clark			University of Washington	Seattle, Washington	Site Contributions	IVY Network member
Karen F.	Miller			Vanderbilt University Medical Center	Nashville, Tennessee	Site Contributions	IVY Network member
Jakea	Johnson			Vanderbilt University Medical Center	Nashville, Tennessee	Site Contributions	IVY Network member
Kelsey N.	Womack			Vanderbilt University Medical Center	Nashville, Tennessee	Site Contributions	IVY Network member
Jillian	Rhoads			Vanderbilt University Medical Center	Nashville, Tennessee	Site Contributions	IVY Network member
Colleen	Ratcliff			Vanderbilt University Medical Center	Nashville, Tennessee	Site Contributions	IVY Network member

*First name, last name, and suffix (if applicable) are required and will appear in PubMed.

***First Name and Middle Initial(s)**	***Last Name**	***Suffix (eg, Jr, III)**	Academic Degrees	Institution	Location (city, state/province, country)	Role or Contribution, eg, chair, principal investigator	Group (if more than 1 Group listed in the byline) and/or Subgroup (eg, Steering Committee)
Ine	Sohn			Vanderbilt University Medical Center	Nashville, Tennessee	Site Contributions	IVY Network member
Cara	Lwin			Vanderbilt University Medical Center	Nashville, Tennessee	Site Contributions	IVY Network member
Julio	Angulo			Vanderbilt University Medical Center	Nashville, Tennessee	Site Contributions	IVY Network member
Stacy	Batey			Vanderbilt University Medical Center	Nashville, Tennessee	Site Contributions	IVY Network member
Shanice	Cummings			Vanderbilt University Medical Center	Nashville, Tennessee	Site Contributions	IVY Network member
Abby	Fink			Vanderbilt University Medical Center	Nashville, Tennessee	Site Contributions	IVY Network member
Claudia	Guevara			Vanderbilt University Medical Center	Nashville, Tennessee	Site Contributions	IVY Network member
Jennifer	Luther			Vanderbilt University Medical Center	Nashville, Tennessee	Site Contributions	IVY Network member
Rendie	McHenry			Vanderbilt University Medical Center	Nashville, Tennessee	Site Contributions	IVY Network member
Bryan	Peterson			Vanderbilt University Medical Center	Nashville, Tennessee	Site Contributions	IVY Network member
Neekar	Rashid			Vanderbilt University Medical Center	Nashville, Tennessee	Site Contributions	IVY Network member
Wanderson	Rezende			Vanderbilt University Medical Center	Nashville, Tennessee	Site Contributions	IVY Network member
Kalee	Rumfelt			Vanderbilt University Medical Center	Nashville, Tennessee	Site Contributions	IVY Network member
Laura	Short			Vanderbilt University Medical Center	Nashville, Tennessee	Site Contributions	IVY Network member
Kevin	Gibbs			Wake Forest University	Winston-Salem, North Carolina	Site Contributions	IVY Network member
Hannah	Strait			Wake Forest University	Winston-Salem, North Carolina	Site Contributions	IVY Network member
Bijal	Parikh			Washington University	St. Louis, Missouri	Site Contributions	IVY Network member
Carleigh	Samuels			Washington University	St. Louis, Missouri	Site Contributions	IVY Network member
Lucy	Vogt			Washington University	St. Louis, Missouri	Site Contributions	IVY Network member
Caroline	O'Neil			Washington University	St. Louis, Missouri	Site Contributions	IVY Network member
Alyssa	Valencia			Washington University	St. Louis, Missouri	Site Contributions	IVY Network member
Francesca	Yerbic			Washington University	St. Louis, Missouri	Site Contributions	IVY Network member
Olivia	Arter			Washington University	St. Louis, Missouri	Site Contributions	IVY Network member
Akshay	Saluja			Washington University	St. Louis, Missouri	Site Contributions	IVY Network member
Elianora	Ovchiyan			Washington University	St. Louis, Missouri	Site Contributions	IVY Network member
Sachina	Mensah			Washington University	St. Louis, Missouri	Site Contributions	IVY Network member
Kim	Vu			Washington University	St. Louis, Missouri	Site Contributions	IVY Network member
David	McDonald			Washington University	St. Louis, Missouri	Site Contributions	IVY Network member
Regine	Burton			Washington University	St. Louis, Missouri	Site Contributions	IVY Network member
Anirudh	Goyal			Yale University	New Haven, Connecticut	Site Contributions	IVY Network member
Ivan	Valesquez			Yale University	New Haven, Connecticut	Site Contributions	IVY Network member
Arda	Yigitkanli			Yale University	New Haven, Connecticut	Site Contributions	IVY Network member
Kimberly	Manchester			Yale University	New Haven, Connecticut	Site Contributions	IVY Network member
