# Supplementary material for: Estimated Effectiveness of 2024-2025 COVID-19 Vaccination Against Severe COVID-19
Source: JAMA Netw Open. 2026 Feb 3;9(2):e2557415. doi: 10.1001/jamanetworkopen.2025.57415 (PMC12869339; doi:10.1001/jamanetworkopen.2025.57415)
Supplement: Supplement 3. — Data Sharing Statement [file jamanetwopen-e2557415-s003.pdf]

Data Sharing Statement

Data
Data available: No

Additional Information
Explanation for why data not available: No additional data are available.
